# Growth inhibitory effects of miR-221 and miR-222 in non-small cell lung cancer cells

**DOI:** 10.1002/cam4.412

**Published:** 2015-01-30

**Authors:** Ryo Yamashita, Mitsuo Sato, Tomohiko Kakumu, Tetsunari Hase, Naoyuki Yogo, Eiichi Maruyama, Yoshitaka Sekido, Masashi Kondo, Yoshinori Hasegawa

**Affiliations:** 1 Department of Respiratory Medicine, Nagoya University Graduate School of Medicine65 Tsurumai-cho, Showa-ku, Nagoya, 466-8550, Japan; 2 Department of Cancer Genetics, Nagoya University Graduate School of MedicineShowa-ku, Nagoya, 466-8550, Japan; 3 Division of Molecular Oncology, Aichi Cancer Center Research InstituteChikusa-ku, Nagoya, 464-8681, Japan

**Keywords:** Apoptosis, cell cycle, epithelial–mesenchymal transition, lung neoplasms, microRNAs

## Abstract

Both pro- and anti-oncogenic roles of miR-221 and miR-222 microRNAs are reported in several types of human cancers. A previous study suggested their oncogenic role in invasiveness in lung cancer, albeit only one cell line (H460) was used. To further evaluate involvement of miR-221 and miR-222 in lung cancer, we investigated the effects of miR-221 and miR-222 overexpression on six lung cancer cell lines, including H460, as well as one immortalized normal human bronchial epithelial cell line, HBEC4. miR-221 and miR-222 induced epithelial-to-mesenchymal transition (EMT)-like changes in a minority of HBEC4 cells but, unexpectedly, both the microRNAs rather suppressed their invasiveness. Consistent with the prior report, miR-221 and miR-222 promoted growth in H460; however, miR-221 suppressed growth in four other cell lines with no effects in one, and miR-222 suppressed growth in three cell lines but promoted growth in two. These are the first results to show tumor-suppressive effects of miR-221 and miR-222 in lung cancer cells, and we focused on clarifying the mechanisms. Cell cycle and apoptosis analyses revealed that growth suppression by miR-221 and miR-222 occurred through intra-S-phase arrest and/or apoptosis. Finally, lung cancer cell lines transfected with miR-221 or miR-222 became more sensitive to the S-phase targeting drugs, possibly due to an increased S-phase population. In conclusion, our data are the first to show tumor-suppressive effects of miR-221 and miR-222 on lung cancer, warranting testing their potential as therapeutics for the disease.

## Introduction

MicroRNAs (miRNAs) are small (about 22 nucleotides long), noncoding, double-stranded RNA molecules that can regulate the expression of target genes 1,2. These miRNAs regulate gene expression by hybridizing to complementary sequences in the 3′ untranslated region (3′ UTR) of target messenger RNA (mRNA). They repress translation of mRNA in part by increasing the instability of mRNAs and inhibit the protein translation by degradation of mRNA 2,3. A number of miRNAs function as oncopromotive or oncosuppressive miRNAs by inhibiting tumor suppressor genes or oncogenes, respectively. Importantly, many investigators reported that suppressing oncopromotive miRNAs and introducing oncosuppressive miRNAs has tumor-suppressive effects 4, suggesting the potential of miRNAs as therapeutic targets or therapeutic agents.

miR-221 and miR-222 are encoded in tandem from a gene cluster located on chromosome Xp11.3 5. Both tumor-suppressive and oncogenic roles of miR-221 and miR-222 have been reported, suggesting they have a bimodal function in the tumorigenesis of human cancers. Oncogenic roles of miR-221 and miR-222 have been shown in several types of human malignancies, especially in breast cancer 6–9. miR-221 and miR-222 exert their oncogenic abilities in part through suppressing the cyclin-dependent kinase (CDK) inhibitors *p27*^*Kip1*^, and *p57* or upregulating the epithelial-to-mesenchymal transition (EMT)-inducing gene *ZEB2* through TRPS1 10–12. On the other hand, several studies reported tumor-suppressive functions of miR-221 and miR-222. One paper reported that overexpression of miR-221 and miR-222 in malignant glioblastoma cells increases the population of cells in S-phase, resulting in massive apoptosis 13. In addition, another paper reported that miR-221 and miR-222 are downregulated in Kaposi sarcoma-associated herpes virus-associated cancers, including primary effusion lymphoma and Kaposi sarcoma 14. Furthermore, a recent study reported that miR-221 enhances the chemosensitivity of cholangiocarcinoma cells to gemcitabine 15.

Garofalo et al. demonstrated that miR-221 and miR-222 play oncogenic roles in lung cancer in part through suppressing the expression of PTEN and TIMP3 tumor suppressor genes 16. To analyze effects of overexpression of miR-221 or miR-222 on invasiveness in lung cancer cells they used one lung cancer cell line, H460, to represent lung cancer cells. However, lung cancer is known to be one of the most genomically diverse of all human cancers 17,18, and therefore in order to obtain more generalized information on how miR-221 and -222 are involved in the pathogenesis of lung cancer, a study using larger panel of lung cancer cell lines is required.

Thus, in the present study, we investigated the effects of miR-221 and miR-222 mimics on six lung cancer cell lines with diverse molecular alterations (three epidermal growth factor receptor (*EGFR*) mutant and three *EGFR* wild type cell lines) as well as one *Cdk4/hTERT*-immortalized normal human bronchial epithelial line, HBEC4 19–22. Consistent with a prior report 16, miR-221 and miR-222 enhanced growth in H460. However, in other five cell lines, effects of miR-221 and miR-222 on growth showed significant differences between cell lines; miR-221 suppressed growth in four cell lines with no effects in one, and miR-222 suppressed growth in three but promoted growth in two. We further show that growth inhibition in lung cancer cell lines by miR-221 and miR-222 overexpression occurred in part through intra-S cell cycle arrest and/or apoptosis. In addition, we suggested that the intra-S arrest was triggered in part by DNA damage as measured by DNA double-strand breaks (DSB). Finally, introduction of miR-221 and miR-222 caused H1299 lung cancer cells, which did not exhibit apoptosis upon introduction of miR-221 and miR-222, to become more sensitive to S-phase targeting drugs, cisplatin, and gemcitabine.

In addition to reported oncogenic functions of miR-221 and miR-222, our findings indicate that they also function as tumor suppressors and can enhance chemosensitivity to cytotoxic drugs, suggesting their potential as therapeutics for lung cancer.

## Material and Methods

### Cell cultures

The 22 lung cancer cell lines and one *Cdk4/hTERT*-immortalized normal human bronchial epithelial cell line, HBEC4 were purchased from ATCC (Manassas, VA) or obtained from the Hamon Center collection (University of Texas Southwestern Medical Center, Dallas, TX) 19–22. Six cell lines including three EGFR-wild-type cell lines (H460, H838, H1299) and three EGFR-mutant cell lines (H3255, HCC4006, HCC4011) were selected for being used for functional analyses. The lung cancer cell lines were cultured in RPMI-1640 (Sigma-Aldrich, St. Louis, MO) supplemented with 10% fetal bovine serum, and HBEC4 was cultured in KSFM (Life Technologies, Gaithersburg, MD) supplemented with 50 ng/mL bovine pituitary extract and 5 ng/mL epidermal growth factor. The authenticity of H838, H1299, H3255, HCC4006, and HCC4011 was confirmed by short tandem repeat analysis. We cultured H460 less than 6 months after obtaining from ATCC and thus did not confirm its authenticity according to the journal policy.

### RNA isolation and quantitative real-time PCR analysis

For mRNA analysis, 5 *μ*g of total RNA isolated using Trizol (Invitrogen Corp., Carlsbad, CA) was reverse transcribed with Superscript III First-Strand Synthesis System using a Random Primer System (Invitrogen). Quantitative reverse transcription PCR (qRT-PCR) analysis of *p27*^*Kip1*^, and *p57* was performed as described previously using the standard Taqman Assay-on-demand PCR protocol 23. We used GAPDH (Applied Biosystems Assay-on demand Life Technologies, Gaithersburg, MD) for mRNA analysis and U6 small nuclear (sn) RNA for microRNA analysis as internal controls.

### Microarray expression analysis

DNA microarray analysis was done using a 3D-Gene Human Oligo chip 25 k (25,370 distinct genes) (Toray Industries, Tokyo, Japan) as described previously 24.

### Western Blot analysis

Western blot analysis was done as described previously using whole cell lysates 25. Primary antibodies used were mouse monoclonal anti-E-CADHERIN (BD Transduction Laboratories, Franklin Lakes, NJ), mouse monoclonal anti-SIP1(ZEB2) (BD Transduction Laboratories), rabbit monoclonal, anti-SLUG (Cell Signaling Technology, Boston, MA), rabbit polyclonal anti-actin (Sigma-Aldrich), rabbit polyclonal anti-cleaved caspase-3, rabbit monoclonal anti-Chk1, rabbit monoclonal anti-phospho-Chk1(Ser317), rabbit monoclonal anti-Chk2, and rabbit monoclonal anti-phospho-Chk2 (Thr68) (all, Cell Signaling Technology). Actin protein levels were measured as a control for equality of protein loading. Anti-rabbit antibody (GE Healthcare, Tokyo, Japan) was used at 1:2000 dilution as a secondary antibody.

### Transfection of MicroRNA mimic

4 × 10^5^ of cells were plated in 10 cm^2^ plates. The next day, cells were transiently transfected with either 10 nmol/L predesigned microRNA mimics (hsa-miR-221 and hsa-miR-222) or control microRNA (microRNA control, AC/eGFP) purchased from Cosmo Bio (Tokyo, Japan), using Lipofectamine RNAiMAX (Invitrogen) according to the manufacturer's protocol. After 24 or 48 h, the transfected cells were harvested for subsequent analyses or were replated for cell growth assays.

### Immunofluorescence staining

Immunofluorescence staining was done as described previously 25. For E-CADHERIN and VIMENTIN staining, mouse monoclonal anti- E-CADHERIN (BD Transduction Laboratories) and mouse monoclonal anti- VIMENTIN (BD Pharmingen, Franklin Lakes, NJ) were used as primary antibodies, respectively. For phospho-histone H2AX staining, rabbit monoclonal anti-phospho-histone H2AX (Cell Signaling Technology) was used as a primarily antibody and Alexa Fluor 488 goat anti-rabbit IgG (1:1000, Molecular Probes, Invitrogen, Gaithersburg, MD) was used as a secondary antibody. 4′,6-diamidino-2-phenylindole (DAPI) solution (Dojindo, Kumamoto, Japan) and rhodamine phalloidin (Molecular Probes, Invitrogen) were used for nucleus and actin staining, respectively, according to the manufacturer's protocols. All stained cells were visualized using an inverted microscope IX73 (Olympus, Tokyo, Japan) with a 20X objective.

### Cell growth assays

A colorimetric proliferation assay was performed using a WST-1 assay kit (Roche, Basel, Switzerland) according to the manufacturer's instructions. Liquid colony formation and soft agar colony formation assays were done as described previously 22.

### BrdU cell proliferation assay

We used a Cell Proliferation ELISA with BrdU (chemiluminescent) kit (Roche) for measurement of BrdU incorporation during DNA synthesis in proliferating cells according to the manufacturer's instructions.

### Cell cycle analysis

Cells were harvested 48 h after transfection of microRNA mimics. Cells were fixed, treated with RNase A, stained with propidium iodide using BD Cycletest Plus Reagent Kit (BD Bioscience, Franklin Lakes, NJ) according to the instructions of the manufacturer, and analyzed for cell cycle status using an FACSCalibur flow cytometer (Becton Dickinson) with BD CellQuest™Pro Ver6 (BD Bioscience, Franklin Lakes, NJ).

### Drug sensitivity assay

Cells were transfected with microRNA mimics (hsa-miR-221 and hsa-miR-222) or control microRNA (microRNA control, AC/eGFP). Forty-eight hours after transfection, cells were seeded in 96-well plates at a density of 2 × 10^4^ cells/mL (50 *μ*L/well) and incubated for 24 h. Then, the cells were treated with various concentrations of cisplatin, gemcitabine, or paclitaxel, for 5 days, and cell viability was measured by a WST-1 assay.

### In vitro invasion assay

Invasion was assayed as described previously using 24-well BD BioCoat Matrigel Invasion Chambers (BD Biosciences) according to the manufacturer's instructions 26.

### Statistical analysis

IBM SPSS ver.21 software (International Business Machines Corp., Armonk, NY) was used for all statistical analyses in this study. The Mann–Whitney *U*-test was used to analyze differences between two groups.

## Results

### miR-221 and miR-222 suppress invasiveness in immortalized normal bronchial epithelial cells

A prior study reported that miR-221 and miR-222 induced EMT in immortalized normal mammary epithelial cells through targeting TRPS1, which inhibits an EMT-inducing transcription factor, ZEB2 9. In addition, another study showed that miR-221 and miR-222 conferred invasiveness on a lung cancer cell line, suggesting that they function as onco-miRNAs in lung cancer cells 16. These findings prompted us to examine whether miR-221 and miR-222 are able to induce EMT in normal lung epithelial cells, and if so, to further test whether the induced- EMT is associated with acquisition of invasiveness or anchorage-independent growth, both well acknowledged EMT-associated malignant phenotypes 27.

**Figure 1:**
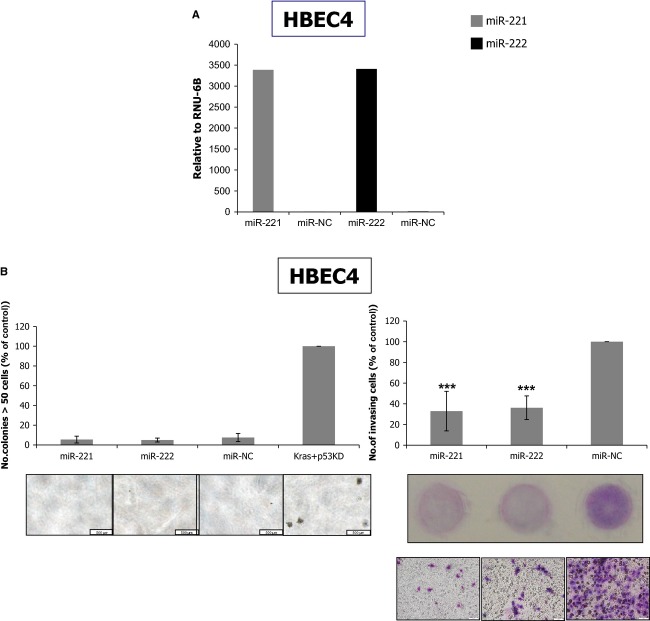
miR-221 and miR-222 suppress invasiveness in immortalized normal bronchial epithelial cells. (A) Quantitative reverse transcription PCR (qRT-PCR) analysis of miR-221 and miR-222 in HBEC4 transfected with miR-221 or miR-222 mimics. After transfection, high expression of these microRNAs was confirmed. (B) Soft agar colony formation (left) and invasion (right) assays for HBEC4 cells transfected with miR-221 or miR-222 mimics. miR-221 and miR-222 did not affect anchorage-independent growth but suppressed invasiveness in HBEC4 cells. The results are averages of three independent experiments done in triplicate wells. ***P < 0.001 (Mann–Whitney *U* test). Kras+p53 knockdown (KD) HBEC4 cells were generated by introducing pLenti6-KRAS^V12^ and pSRZ-p53 21,22 vectors into HBEC4 cells and were used as positive control for the soft agar colony formation assay.

To this end, we used the HBEC4, which is one of a series of HBEC lines we established by introducing *hTERT* and *Cdk4* into normal bronchial epithelial cells obtained from a noncancerous lesion of the lung 20 as a model, because HBEC cells have epithelial phenotypes and do not have malignant phenotypes that include invasiveness and anchorage-independent growth. After introduction of miR-221 and miR-222 mimics into HBEC4, increased expression of these miRNAs was confirmed by qRT-PCR analysis (Fig.1A). These increased levels of miR-221 and miR-222 were much higher than those endogenously expressed in a panel of lung cancer cell lines (Fig. S1). Forty eight hours after the transfection of miR-221 and miR-222, a minority of cells underwent morphologic changes suggestive of EMT including switch to elongated spindle shape (Fig. S2). After the introduction of miR-221 or miR-222, protein expression of E-CADHERIN, a hallmark epithelium marker did not significantly change in both Western blot and immunocytochemistry while protein expression of VIMENTIN, a marker for mesenchymal cells, increased in immunocytochemistry (Figs. S3 and S4). Expression of two, EMT-inducing transcription factors, SIP1 (ZEB2), shown to be upregulated by miR-221 and miR-222 in mammary epithelial cells 9 and SLUG remain unchanged. These results suggested that miR-221 and miR-222 only modestly caused immortalized normal bronchial epithelial cells to undergo EMT.

Next, we assessed whether these modest EMT-like changes were associated with anchorage-independent growth or invasiveness, both well-acknowledged EMT-associated oncogenic phenotypes 27. Anchorage-independent growth, as measured by growth in soft agar, was not affected by miR-221 or miR-222 (Fig.1B, left). Invasiveness was rather suppressed by miR-221 and miR-222 (Fig.1B, right). These results indicated that miR-221 and miR-222 induced EMT-like changes to a small extent in normal lung epithelial cells but did not enhance the EMT-associated phenotypes of anchorage-independent growth or invasiveness. miR-221 and miR-222 rather suppressed invasiveness in these cells, suggesting their tumor-suppressive roles in normal bronchial epithelial cells.

### Effects of miR-221 and miR-222 on growth differ strikingly between lung cancer cell lines

**Figure 2:**
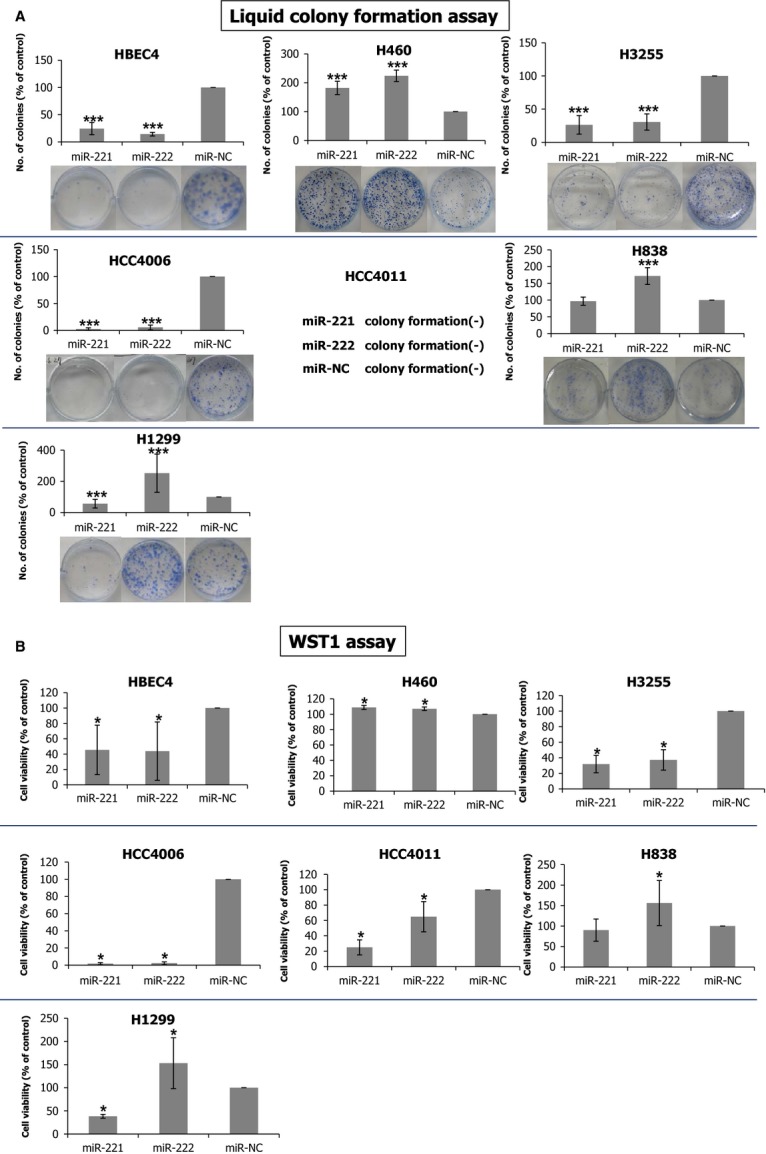
Effects of miR-221 and miR-222 on growth differ strikingly between lung cancer cell lines. Liquid colony formation assay (A) and WST-1 colorimetric proliferation assay (B) for six lung cancer cell lines and HBEC4 transfected with miR-221 or miR-222 mimics. The results are averages of three independent experiments done in triplicate wells (liquid colony assay) and octuplicate wells (WST-1 assay). *, and *** indicate *P* < 0.05, and *P* < 0.001 (Mann–Whitney *U* test), respectively.

We evaluated the effects of miR-221 and miR-222 mimics on cell growth in six lung cancer cell lines (H460, H3255, HCC4006, HCC4011, H838, H1299) and HBEC4. Efficient introduction of miR-221 and miR-222 into these cell lines was confirmed by qRT-PCR analysis (Fig. S5). Cell growth was evaluated by a WST-1 colorimetric proliferation assay and liquid colony formation assay. Consistent with a prior report 16 miR-221 and miR-222 promoted growth in H460. Nevertheless, unexpectedly, the effects of miR-221 and miR-222 introduction in other five cancer cell lines significantly differed between cell lines. miR-221 suppressed growth in four cell lines (H3255, HCC4006, HCC4011, H1299) without affecting growth in one (H838), and miR-222 suppressed growth in three (H3255, HCC4006, HCC4011) but promoted growth in two (H838, H1299) (Fig.2A and B).

To gain insight into possible molecular mechanisms underlying the striking difference in the responses to introduction of miR-221 and miR-222, especially to miR-222, between lung cancer cell lines, we performed microarray gene expression analysis on H3255, representing cell lines whose growths were suppressed by miR-222, and H1299, representing cell lines whose growth were promoted by miR-222, using a 3D-Gene Human Oligo chip 25 k (Toray Industries). Upon introduction of miR-221 or miR-222, numerous genes were up- or downregulated, including several known target genes such as *p27*^*Kip1*^*, p57, PTEN, Dicer1,* and *APAF1* (Figs. S6, S7, S8, and S9). Expression levels of each gene differentially expressed by miR-221 strongly correlated with those of miR-222 in both H1299 and H3255 (Spearman correlation coefficient values: 0.983 (*P* < 0.001) for H1299 and 0.987 (*P* < 0.001) for H3255) (Figs. S10 and S11). Pathway analysis using NIH-DAVID 28,29, a web interface functional annotation tool, revealed that several pathways were significantly affected by miR-221 or miR-222 (Tables S1, S2, S3, and S4) in H1299 and H3255. The pathways affected by miR-221 greatly overlapped with those affected by miR-222 in H3255, while overlapping of only one pathway (Melanoma) was seen in H1299, which may be responsible for the opposite effects of miR-221 and miR-222 seen in H1299, but not in H3255. Recently, by doing pathway analysis Kneitz et al. found that three apoptosis-related pathways are activated in miR-221-transfected prostate cancer cells, including the Toll-like receptor, the RIG-like receptor, and the JAK/STAT pathways 30. We also found that all the three pathways are significantly affected in both miR-221 and miR-222 treated H3255 cells but not in H1299 cells, suggesting that these pathways could contribute to the apoptosis induced by miR-221 or miR-222 in H3255 cells.

### Growth suppression by miR-221 and miR-222 in lung cancer cells occurs through intra-S arrest and/or apoptosis

**Figure 3:**
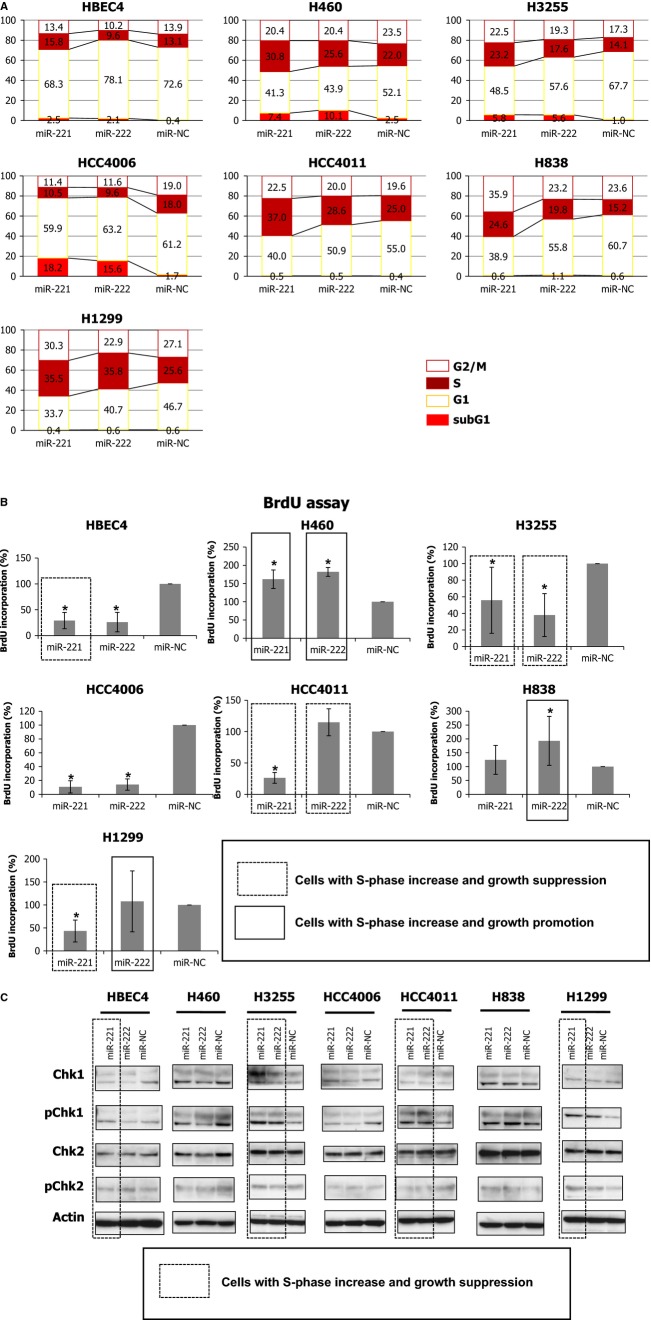
miR-221 and miR-222 suppress growth in lung cancer cells through intra-S arrest and/or apoptosis. (A) Distribution of cell cycle phases of six lung cancer cell lines and HBEC4 transfected with miR-221 or miR-222 mimics by flow cytometry. Numerical values in the figure are averages of three independent experiments. (B) BrdU incorporation in six lung cancer cell lines and HBEC4 transfected with miR-221 or miR-222 mimics. The results are averages of three independent experiments done in octuplicate. *indicates *P* < 0.05 (Mann–Whitney *U* test). Six cases in which cells exhibit both an increase in S-phase by FACS analysis and growth suppression in liquid colony formation analysis are enclosed by a dotted line and in five of these six cases BrdU incorporation are decreased. Cells exhibit both an increase in S-phase and growth promotion are enclosed by a solid line. (C) Western blots of Chk1, phosphorylated Chk1 (pChk1), Chk2, and phosphorylated Chk2 (pChk2) for lung cancer cell lines and HBEC4 transfected with miR-221 or miR-222 mimics. Six cases in which cells exhibit both an increase in S-phase by FACS analysis and growth suppression in liquid colony formation analysis are enclosed by a dotted line and in five of the six cases pChk1 or pChk2 levels were increased.

To elucidate the molecular mechanisms of miR-221- and miR-222-induced growth suppression in Non-small cell lung cancer (NSCLC) cells, we performed cell cycle and apoptosis analyses. Cell cycle analysis revealed that introduction of miR-221 and miR-222 resulted in increased S-phase populations in H460, H3255, HCC4011, H838, and H1299 cell lines (Fig.3A and Fig. S12). miR-221 tended to increase S phase populations to a greater extent than miR-222 did. Introduction of miR-221 and miR-222 resulted in increases in sub-G1 populations, which correspond to apoptotic cells, in H460, H3255 and HCC4006. The apoptosis in these three cell lines was also confirmed by Western blot of cleaved caspase-3 (Fig. S13).

A previous study reported that introduction of miR-221 and miR-222 resulted in downregulation of p27^*Kip1*^ and p57, regulators of the G1/S transition, leading to premature S-phase entry, and subsequent apoptosis in glioblastoma cells 13. To see whether miR-221 and miR-222-induced apoptosis in NSCLC cells occurs through the same mechanism as in glioblastoma, we examined whether miR-221 and miR-222 suppressed *p27*^*Kip1*^ and *p57* expression. We found that miR-221 and miR-222 only occasionally suppressed *p27*^*Kip1*^ and *p57* expression in NSCLC cells (Fig. S14), suggesting that suppression of *p27*^*Kip1*^ and *p57* expression may not be solely responsible for impaired G1/S checkpoints that lead to the increased S-phase population in cells transfected with miR-221 or miR-222 in NSCLC cells. Next, to investigate whether the increased S-phase population reflects an increase in DNA synthesis or arrest in the intra-S-phase, we analyzed BrdU incorporation and found that in five of six cases where cells transfected with miR-221 or miR-222 exhibited both S-phase increases in FACS analysis and growth suppression in liquid colony formation assay (enclosed by a dotted line in Fig.3B) showed decreased BrdU incorporation (Fig.3B). This suggested that the increase in S-phase observed in cells showing growth suppression reflected intra-S-phase arrest. Nevertheless, we realized that we were unable to conclusively state this finding because we did not perform FACS analysis with simultaneous double staining of DNA content and BrdU that enables evaluating BrdU incorporation exclusively in S-phase cells. By contrast, in cells showing increase in S-phase and growth promotion by miR-221 or miR-222 exhibited increased BrdU incorporation, suggesting that in these cells S-phase increase reflected increased replication of DNA (enclosed by a solid line in Fig.3B). Furthermore, we examined the levels of phosphorylated Chk1 and Chk2 (pChk1 and pChK2), markers of intra-S phase arrest 31. In all cases except for miR-221-transfected-HBEC4, when cells transfected with miR-221 or miR-222 showed both increased S-phase population in FACS analysis and growth suppression in liquid colony formation assay (enclosed by a dotted line in Fig.3C), pChk1 and/or pChk2 levels were upregulated, suggesting that the S-phase increase due to miR-221 or miR-222 reflected intra-S phase arrest (Fig.3C).

**Figure 4:**
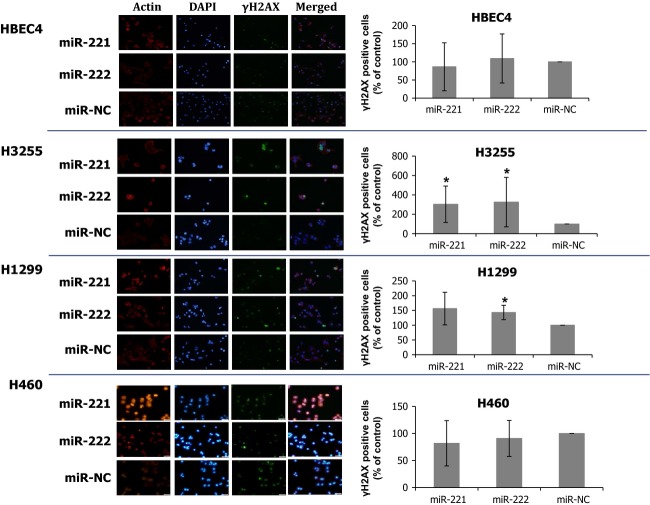
Introduction of miR-221 and miR-222 results in increase in *γ*H2AX-positve cells in lung cancer cell lines. miR-221 and miR-222 introductions increased the number of H3255 and H1299 cells positive for nuclear *γ*H2AX staining cells. This result suggests that unrepaired DSBs increased upon introduction of miR-221 and miR-222 in these cells. *γ*H2AX positive cells (% of control) are averages of three or four independent experiments. *indicates *P* < 0.05 (Mann–Whitney *U* test).

We further asked what events triggered the intra-S phase arrest in miR-221- and miR-222-transfected lung cancer cells. We hypothesized that G1/S checkpoint impairment by miR-221 and miR-222 resulted in the accumulation of DNA damages, which led to intra-S arrest and further caused cells to undergo apoptosis, depending on the cellular capability for repairing DNA damages. We evaluated the levels of DNA DSB, the most critical DNA damage, which is caused by external insults such as gamma irradiation and cytotoxic drugs or endogenously generated DNA damage 32,33. Unless DNA DSBs are repaired, cells undergo apoptosis at high frequency 33. We examined the expression of *γ*H2AX in the nucleus, a marker for DSB 32,33. miR-221 and miR-222 treatment increased percentages of *γ*H2AX-positve cells in H1299 and H3255 but not in HBEC4 or H460, suggesting the increase in unrepaired DSBs in H1299 and H3255 cells (Fig.4). Nevertheless, *γ*H2AX staining did not exhibit typical foci pattern, usually seen in cells with DSBs, but instead cells were homogeneously stained by *γ*H2AX, and thus, our data only suggest that increased DSBs induced by miR-221 and miR-222 may cause S-phase arrest and apoptosis.

Collectively, these findings showed that miR-221- and miR-222-induced growth suppression in NSCLC cells occurred in part through intra-S phase arrest and/or apoptosis.

### miR-221 and miR-222 enhance chemosensitivities to S-phase targeting drugs, cisplatin, and gemcitabine, but do not affect sensitivities to an M-phase targeting drug, paclitaxel, in lung cancer cells

**Figure 5:**
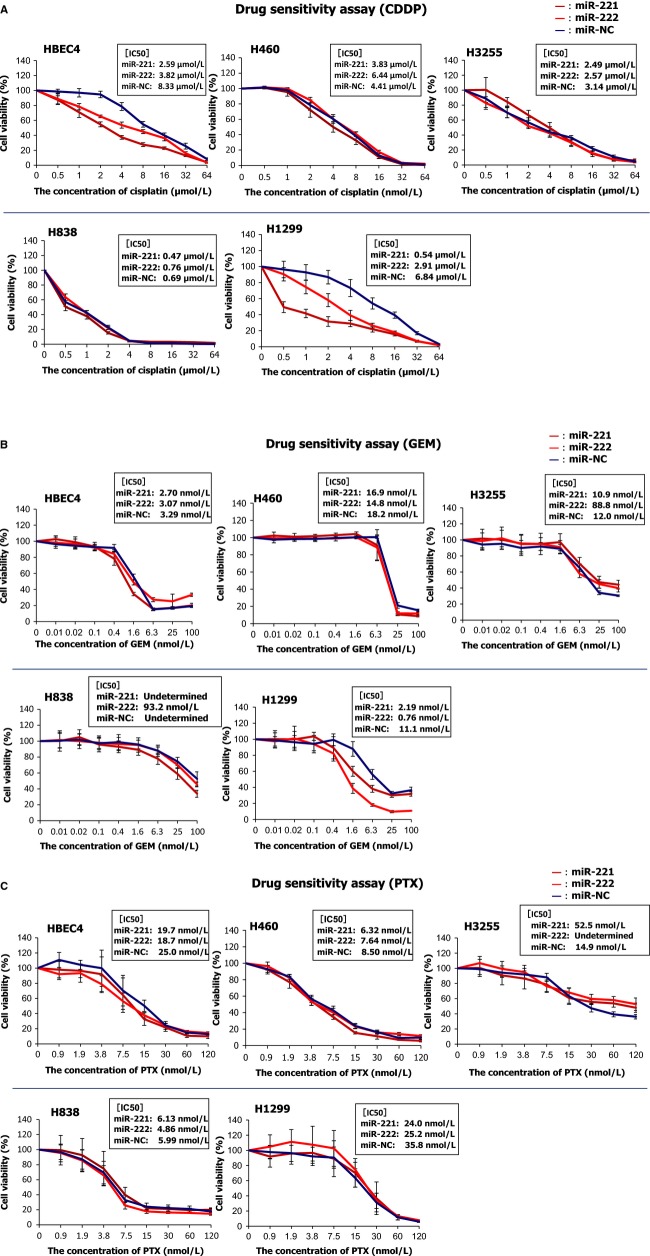
miR-221 and miR-222 enhance chemosensitivities to cisplatin (A) and gemcitabine (B), but do not affect those to paclitaxel (C) in lung cancer cells. Cells were transfected with miR-221 or miR-222 mimics, and 48 h after transfection, they were seeded in 96-well plates at a density of 2 × 10^4^ cells/mL (50 *μ*L/well) and incubated for 24 h. Then the cells were treated with various doses of cisplatin, gemcitabine, or paclitaxel for 5 days, and cell viability was measured by a WST-1 assay. The graphs are representative results of three independent experiments. IC50 values are averages of three independent experiments.

We hypothesized that the increase in S-phase populations by miR-221 and miR-222 improves sensitivities to S-phase targeting drugs in lung cancer cells. We used cisplatin and gemcitabine as S-phase targeting drugs because these drugs are commonly used for the treatment for lung cancer in the clinic 34. We selected four NSCLC cell lines H460, H838, H3255, and H1299. These cell lines showed increased S-phase populations due to miR-221 and miR-222, but they differed in that H460 and H3255, but not H838 or H1299 exhibited apoptosis. Transfection of miR-221 or miR-222 improved sensitivities to cisplatin in H1299 and HBEC4 but not in H460, H3255, or H838 (Fig.5). Improved sensitivities to gemicitabine were seen in H1299 cells transfected with miR-221 or miR-222 mimics, but not seen in other cell lines. By contrast, miR-221 or miR-222 mimics did not enhance sensitivity to the M-phase targeting drug paclitaxel in any cell line studied. These results show that miR-221 and miR-222 increased sensitivities of lung cancer cells to the S-phase targeting drugs cisplatin and gemcitabine in some cases but did not affect sensitivity to the M-phase targeting drug paclitaxel, suggesting their potential as chemosensitizers of S-phase targeting drugs.

## Discussion

In the present study, we examined the effects of miR-221 and miR-222 on growth and drug sensitivity in lung cancer cells. A prior paper reported that miR-221 and miR-222 enhanced invasiveness in H460 16. Consistent with this report, we found that miR-221 and miR-222 promoted growth in H460, while in other five cells lines miR-221 suppressed growth in four cell lines without affecting one and miR-222 suppressed growth in the three cell lines but promoted growth in two. These data were the first to demonstrate growth suppressive effects of miR-221 and miR-222 in lung cancer cells. Growth suppression by miR-221 or miR-222 occurred through S-phase arrest and/or apoptosis in part resulting from DNA DSBs. Finally, our results showed that introduction of miR-221 or miR-222 enhanced sensitivities to the S-phase targeting drugs cisplatin and gemcitabine but did not affect an M-phase targeting drug, paclitaxel. Collectively, our results suggest growth inhibitory effects of miR-221 and miR-222 in lung cancer cells.

Our data exhibit concordance with findings of Medina et al. who reported that introduction of miR-221 and miR-222 impaired the G1/S checkpoint by suppressing p27^*Kip1*^ and p57, leading to a massive increase in the S-phase population and subsequent apoptosis 13. We also observed that introduction of miR-221 and miR-222 resulted in increases in S-phase populations in five of six lung cancer cell lines. However, in our experiments, only two (H460, H3255) of the five lines with S-phase increases underwent apoptosis. The absence of apoptosis in the other cell lines could be explained by the difference in the degree of the S-phase increase. The prior report showed an approximately 40% increase of S-phase after miR-221 or miR-222 introduction 13, while we saw only 5–10% increase. The degree of S-phase increase induced by impaired G1/S transition may be correlated with the severity of resultant DNA damage, and therefore we postulate that the difference in the degree of S-phase increase between cells transfected with miR-221 or miR -222 may influence whether cells undergo apoptosis or not.

On the other hand, our findings showing tumor-suppressive roles for miR-221 and miR-222 seem to contrast with a study reported by Garofalo et al. 16,35. They reported that miR-221 and miR-222 enhanced tumorigenic phenotypes of H460 lung cancer cells, including invasiveness and resistance to TRAIL-induced apoptosis, through suppressing PTEN and TIMP3, both tumor suppressor genes. We used the same cell line, H460 and obtained consistent results showing that both miR-221 and miR-222 promoted liquid colony formation in H460 (Fig.2A). This suggests that it is unlikely that our finding that miR-221 and miR-222 showed tumor-suppressive roles in many lung cancer cell lines is attributable to experimental errors. In addition, to determine whether the growth suppression by miR-221 or miR-222 that we observed in this study coincided with suppression of PTEN, we performed Western blots of PTEN in lung cancer cells transfected with either miRNA and found that not all but in several cases where cell growth was suppressed (e.g., H3255, HCC4006) PTEN expression was significantly suppressed (Fig. S15). This showed that despite the inhibition of PTEN, miR-221 and miR-222 overexpression exert growth suppressive function in certain cellular context.

In conclusion, our results show that the effects of miR-221 and miR-222 introduction on lung cancer cells vary significantly between cell lines, but that they could be attractive therapeutics for lung cancer especially when efficient methods to predict whether they function as a tumor suppressor or not is developed.

## Supporting Information

Figure S1 Expression of miR-221 and miR-222 in non-small cell lung cancer cell lines. qRT-PCR analysis of miR-221 and miR-222 in 22 non-small cell lung cancer and HBEC4 cell lines. The data are averages of two independent PCR reactions. **Figure S2.**EMT-like changes in HBEC4 transfected with miR-221 or miR-222. HBEC4 cells transfected with miR-221 or miR-222 mimics. Upon introduction of miR-221 or miR-222 mimics, a subset of cells changed to elongated spindle shapes, and these morphologic changes suggest the occurrence of EMT. Red arrows indicate cells exhibiting EMT-like morphological changes. **Figures S3 and S4.** Expression of EMT markers in HBEC4 transfected with miR-221 or miR-222. Western blot of E-CADHERIN, SIP1(ZEB2), and SLUG (Fig. S3) and immunocytochemistry of E-CADHERIN and VIMENTIN (Fig. S4) in HBEC4 cells transfected with miR-221 or miR-222 mimics. In Figure S3, the left shows images of immunocytochemistry and the right graphs show percentages of cells positive for E-CADHERIN or VIMENTIN. The results are averages of three independent experiments done in octuplicate. *Indicates *P* < 0.05 (Mann–Whitney *U* test). **Figure S5.**qRT-PCR of miR-221 and miR-222 in lung cancer cell lines transfected with miR-221 or miR-222 mimics. After transfection, high levels of these microRNAs are expressed. **Figure S6.** Scatter plot of differentially expressed genes between H1299 cells transfected with miR-221 and those transfected with control. Blue lines represent twofold change and a red line represents an identity line. **Figure S7.** Scatter plot of differentially expressed genes between H1299 cells transfected with miR-222 and those transfected with control. Blue lines represent twofold change and a red line represents an identity line. **Figure S8.** Scatter plot of differentially expressed genes between H3255 cells transfected with miR-221 and those transfected with control. Blue lines represent twofold change and a red line represents an identity line. **Figure S9.** Scatter plot of differentially expressed genes between H3255 cells transfected with miR-222 and those transfected with control. Blue lines represent twofold change and a red line represents an identity line. **Figure S10.** Scatter plot of genes differentially expressed between H1299 cells transfected with miR-221 and those transfected with miR-222. Blue lines represent twofold change and a red line represents an identity line. **Figure S11.** Scatter plot of genes differentially expressed between H3255 cells transfected with miR-221 and those transfected with miR-222. Blue lines represent twofold change and a red line represents an identity line. **Figure S12**. Flow cytometry of six lung cancer cell lines and HBEC4 transfected with miR-221 or miR-222 mimics. Forty-eight hours after transfection, cells were stained with propidium iodine, and cell cycles of 20,000 cells were profiled. Representative results of three independent experiments are shown, and numerical values in the figure are averages of three independent experiments. **Figure S13.** Western blot of cleaved caspase-3 for lung cancer cell lines and HBEC4 transfected with miR-221 or miR-222- mimics. **Figure S14**. qRT-PCR analysis of *p27*^*Kip1*^ and *p57* in lung cancer cell lines and HBEC4 transfected with miR-221 or miR-222 mimics. The results are averages of two independent PCR experiments done in duplicate. **Figure S15.** Western blot of PTEN in lung cancer and HBEC4 cells transfected with miR-221 or miR-222. *PTEN expression is suppressed by miR-221 or miR-222- mimics.

Table S1 Pathways with significant enrichment for genes more than twofold up- or downregulated after miR-221 introduction in H1299. **Table S2.** Pathways with significant enrichment for genes more than twofold up- or downregulated after miR-222 introduction in H1299. **Table S3.** Pathways with significant enrichment for genes more than twofold up- or downregulated after miR-221 introduction in H3255. **Table S4.** Pathways with significant enrichment for genes more than twofold up- or downregulated after miR-222 introduction in H3255.
