# Supplementary material for: Growth inhibitory effects of miR-221 and miR-222 in non-small cell lung cancer cells
Source: Cancer Med. 2015 Jan 30;4(4):551–64. doi: 10.1002/cam4.412 (PMC4402070; doi:10.1002/cam4.412)
Supplement: Figure S1 — Expression of miR-221 and miR-222 in non-small cell lung cancer cell lines. qRT-PCR analysis of miR-221 and miR-222 in 22 non-small cell lung cancer and HBEC4 cell lines. The data are averages of two independent PCR reactions. Figure S2.EMT-like changes in HBEC4 transfected with miR-221 or miR-222. HBEC4 cells transfected with miR-221 or miR-222 mimics. Upon introduction of miR-221 or miR-222 mimics, a subset of cells changed to elongated spindle shapes, and these morphologic changes suggest the occurrence of EMT. Red arrows indicate cells exhibiting EMT-like morphological changes. Figures S3 and S4. Expression of EMT markers in HBEC4 transfected with miR-221 or miR-222. Western blot of E-CADHERIN, SIP1(ZEB2), and SLUG (Fig. S3) and immunocytochemistry of E-CADHERIN and VIMENTIN (Fig. S4) in HBEC4 cells transfected with miR-221 or miR-222 mimics. In Figure S3, the left shows images of immunocytochemistry and the right graphs show percentages of cells positive for E-CADHERIN or VIMENTIN. The results are averages of three independent experiments done in octuplicate. *Indicates P < 0.05 (Mann–Whitney U test). Figure S5.qRT-PCR of miR-221 and miR-222 in lung cancer cell lines transfected with miR-221 or miR-222 mimics. After transfection, high levels of these microRNAs are expressed. Figure S6. Scatter plot of differentially expressed genes between H1299 cells transfected with miR-221 and those transfected with control. Blue lines represent twofold change and a red line represents an identity line. Figure S7. Scatter plot of differentially expressed genes between H1299 cells transfected with miR-222 and those transfected with control. Blue lines represent twofold change and a red line represents an identity line. Figure S8. Scatter plot of differentially expressed genes between H3255 cells transfected with miR-221 and those transfected with control. Blue lines represent twofold change and a red line represents an identity line. Figure S9. Scatter plot of differentially [file cam40004-0551-sd1.ppt]

## Slide 1
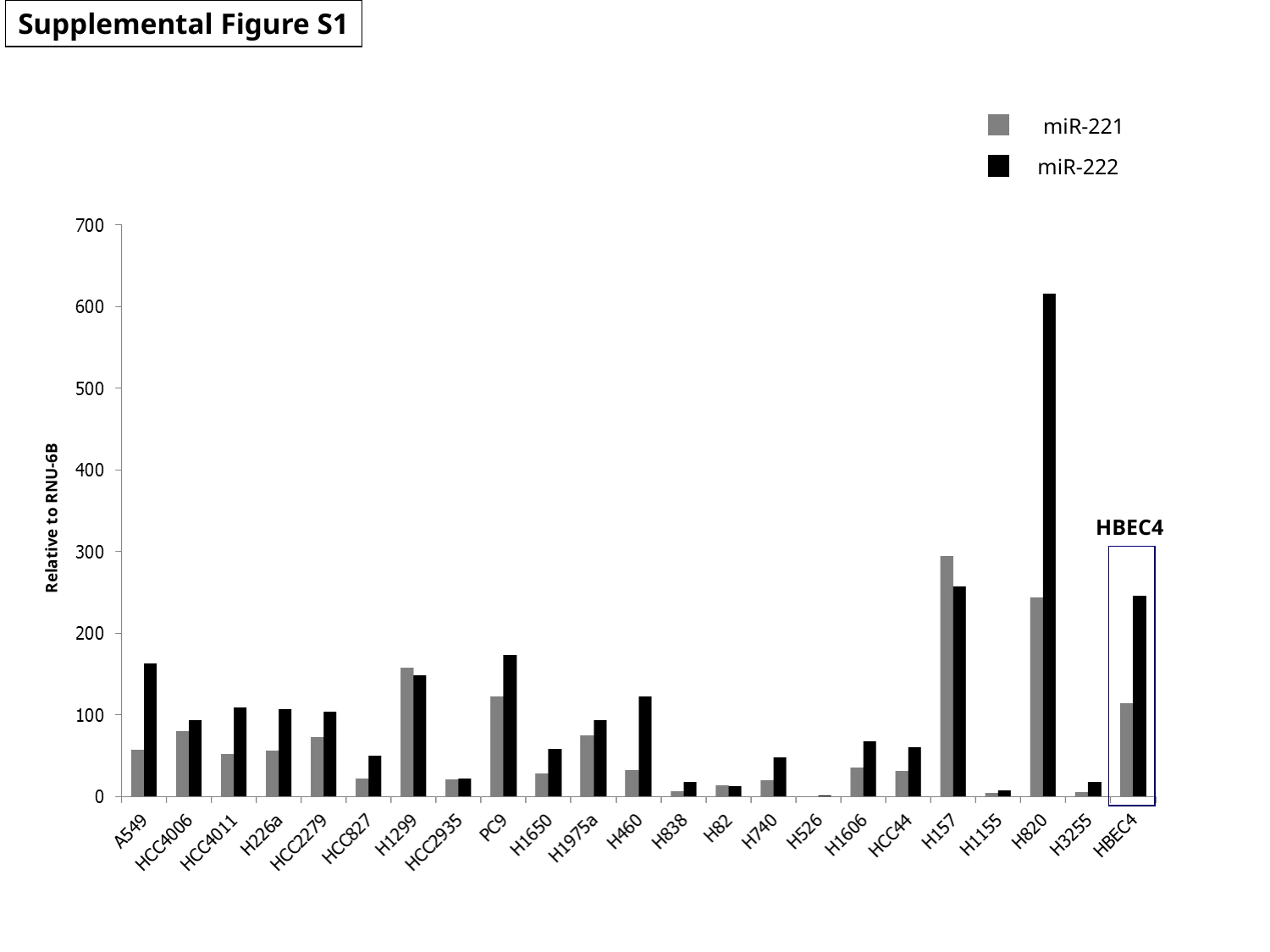

Supplemental Figure S1
 　miR-221
　miR-222
HBEC4
Relative to RNU-6B

## Slide 2
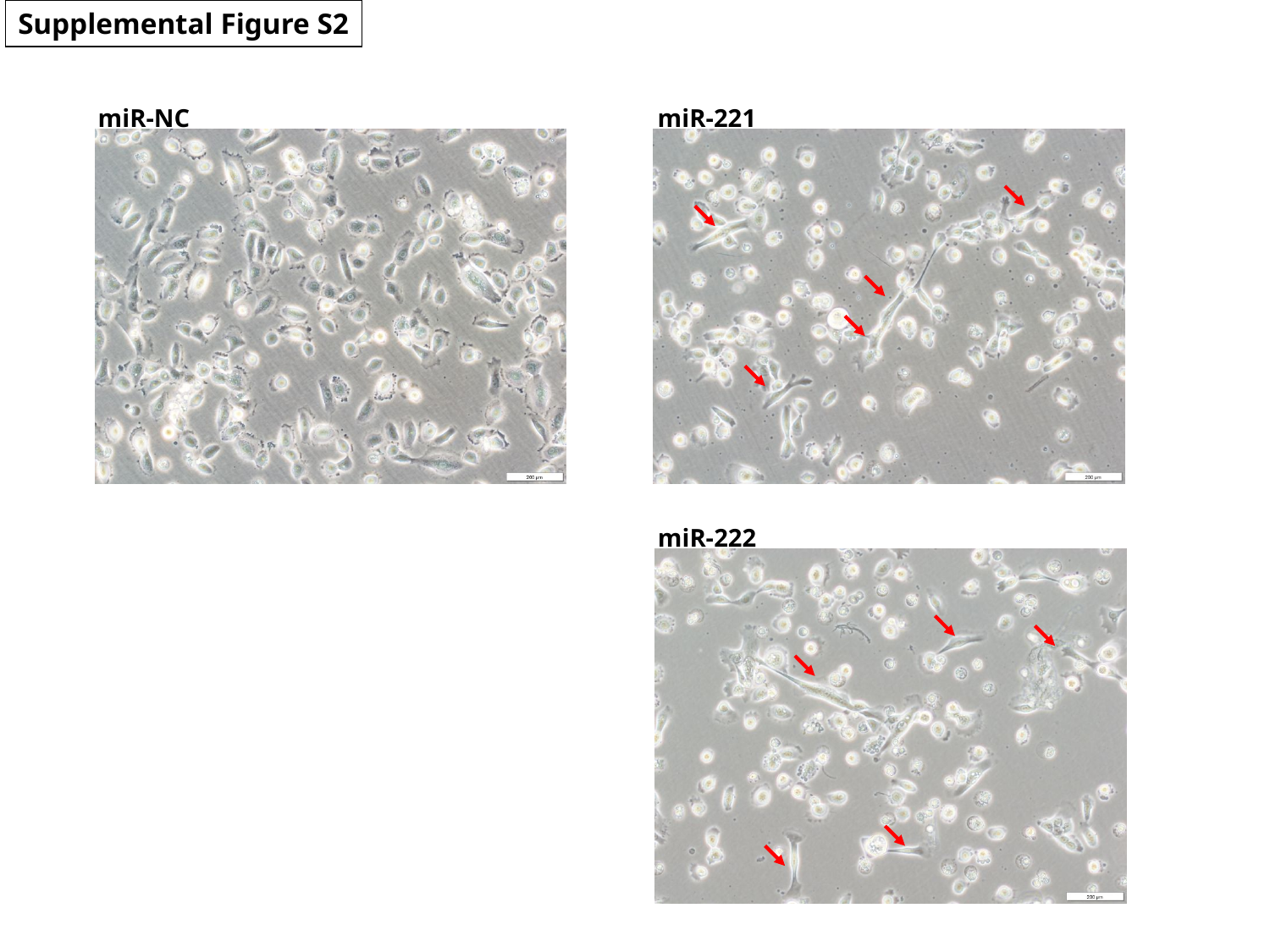

Supplemental Figure S2
miR-NC
miR-221
miR-222

## Slide 3
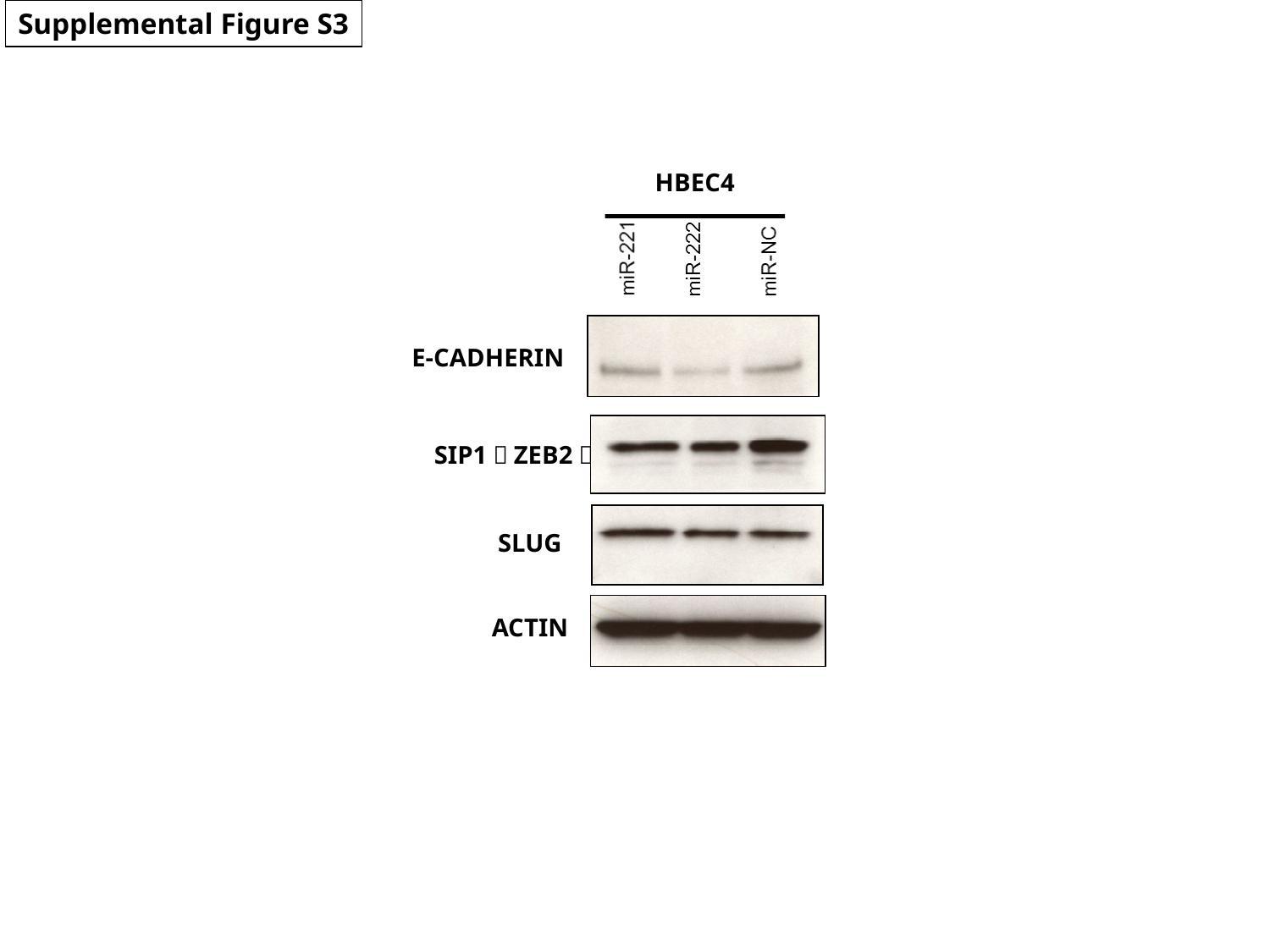

Supplemental Figure S3
HBEC4
E-CADHERIN
SIP1（ZEB2）
SLUG
ACTIN

## Slide 4
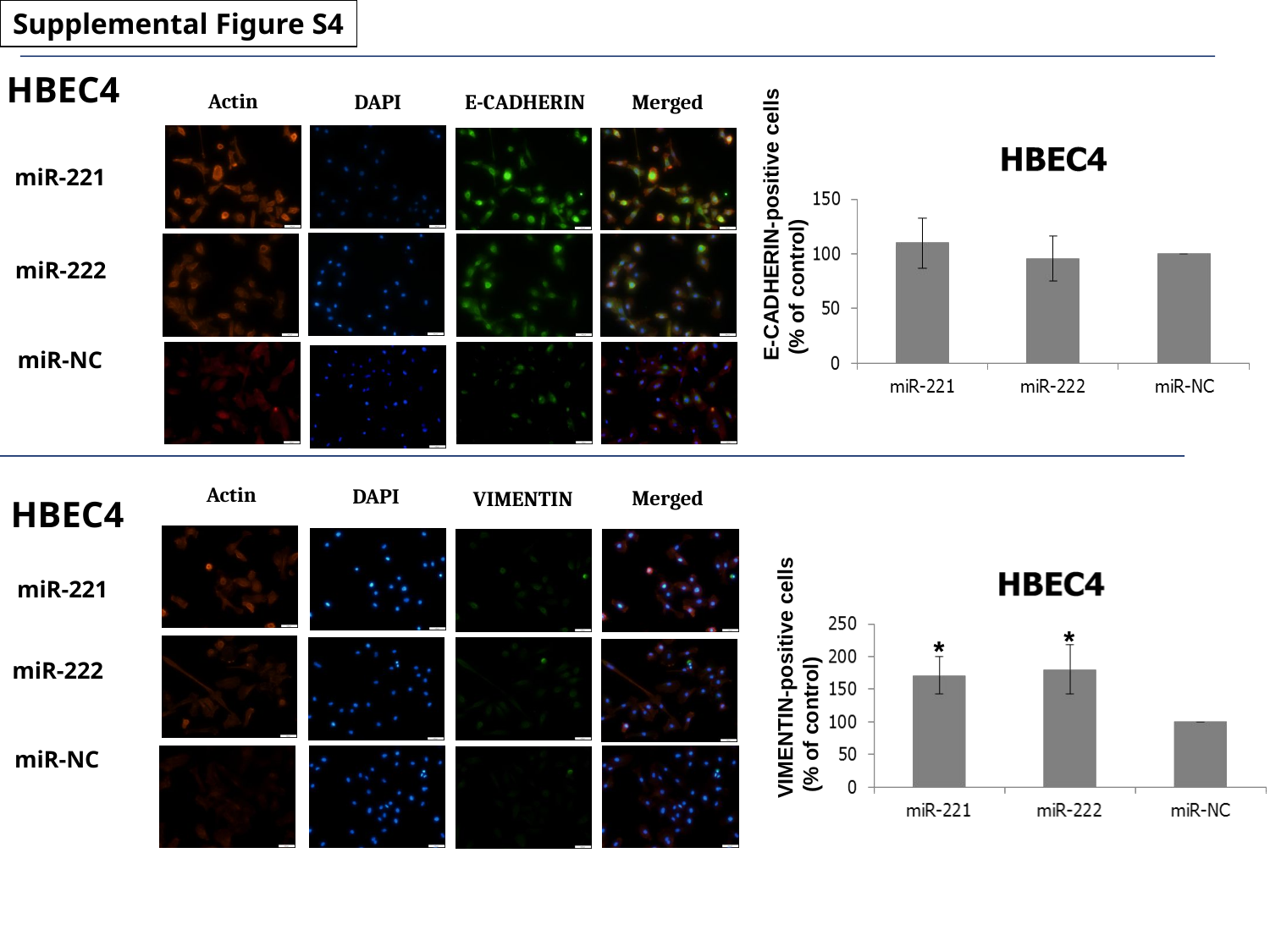

Supplemental Figure S4
HBEC4
DAPI
Actin
Merged
miR-221
miR-222
miR-NC
E-CADHERIN
E-CADHERIN-positive cells
 (% of control)
VIMENTIN
HBEC4
miR-221
miR-222
miR-NC
DAPI
Actin
Merged
*
*
VIMENTIN-positive cells
 (% of control)

## Slide 5
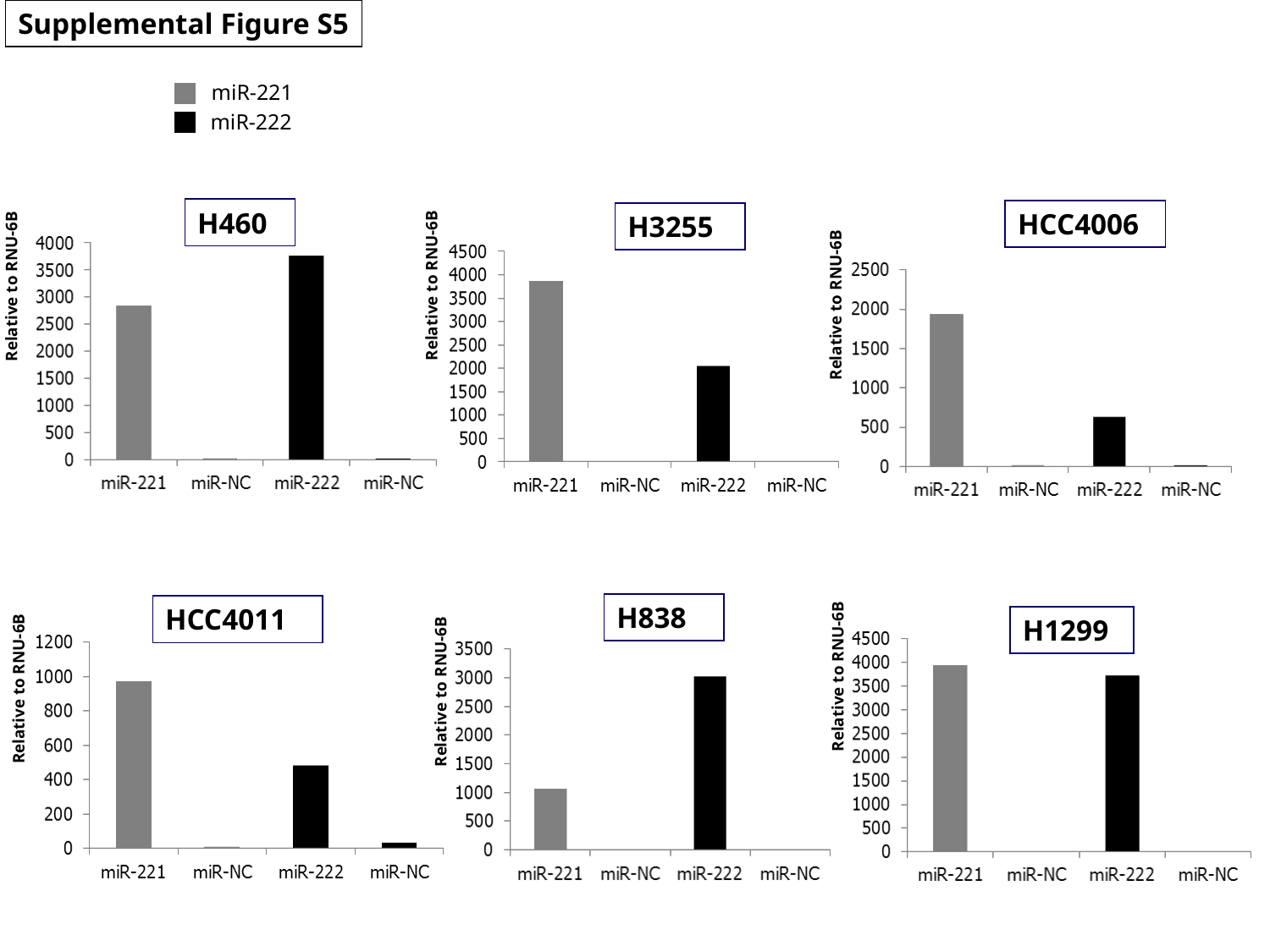

Supplemental Figure S5
 miR-221
miR-222
H3255
Relative to RNU-6B
Relative to RNU-6B
H460
HCC4006
Relative to RNU-6B
H1299
Relative to RNU-6B
H838
Relative to RNU-6B
HCC4011
Relative to RNU-6B

## Slide 6
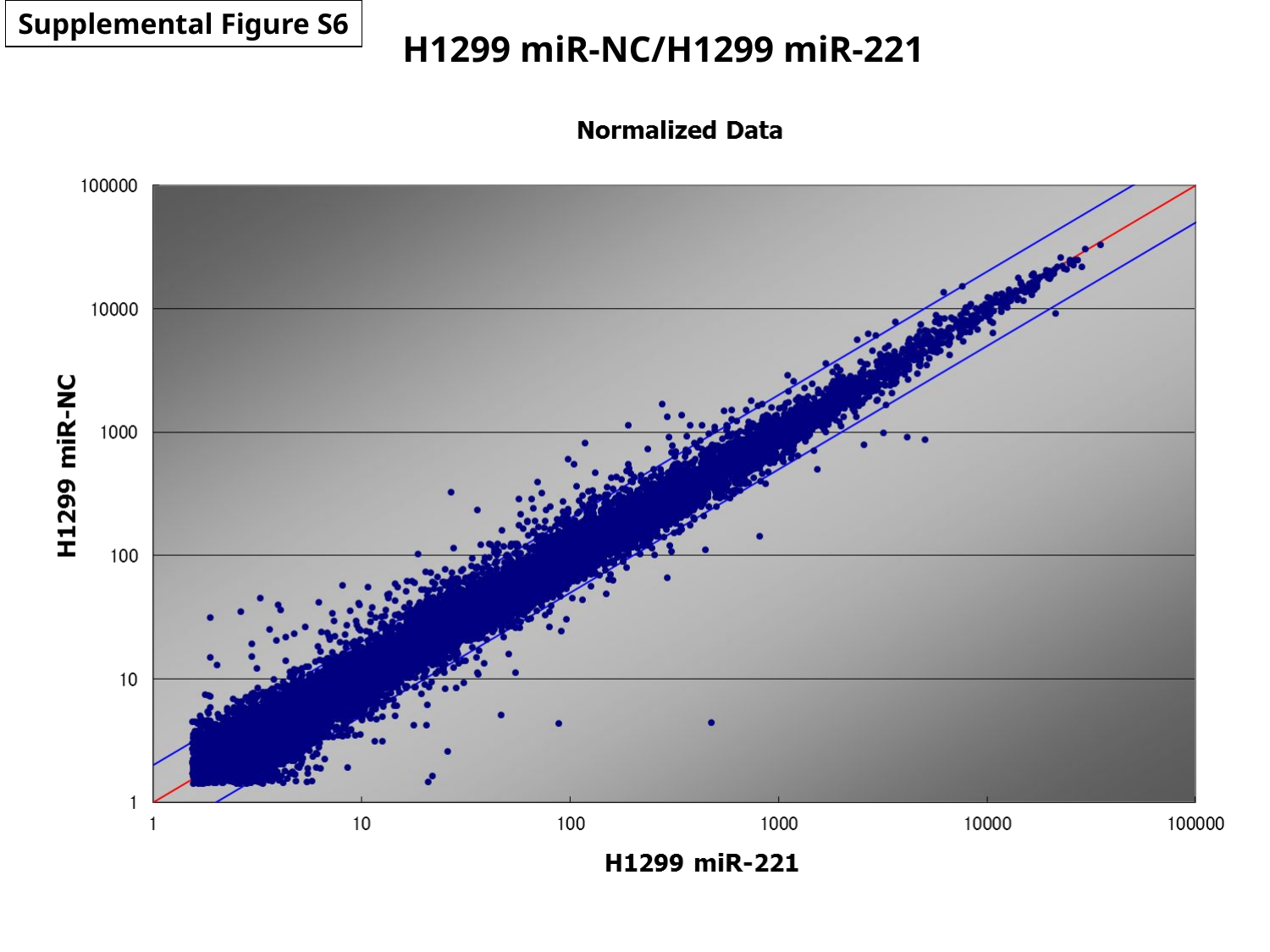

Supplemental Figure S6
# H1299 miR-NC/H1299 miR-221

## Slide 7
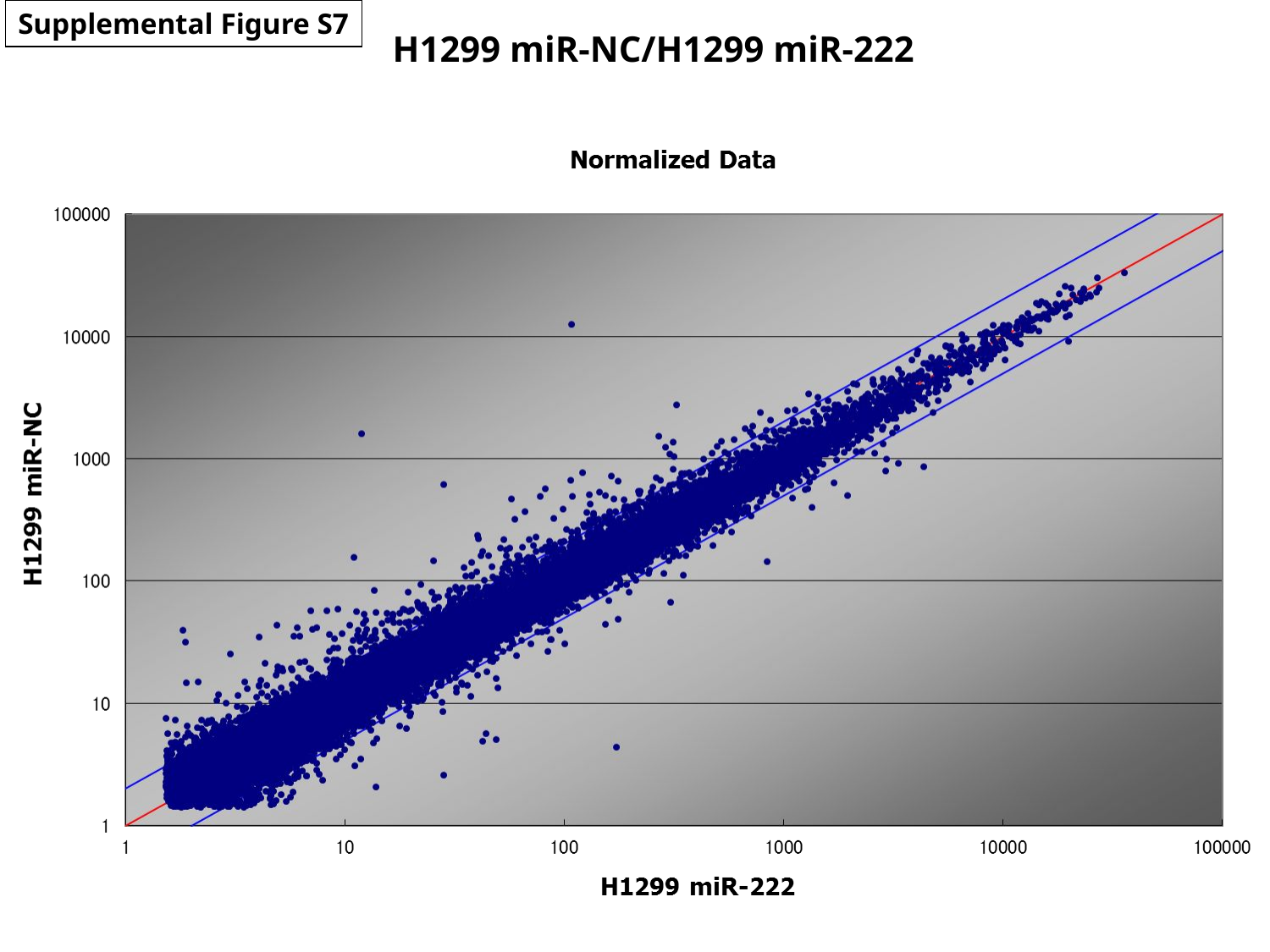

Supplemental Figure S7
# H1299 miR-NC/H1299 miR-222

## Slide 8
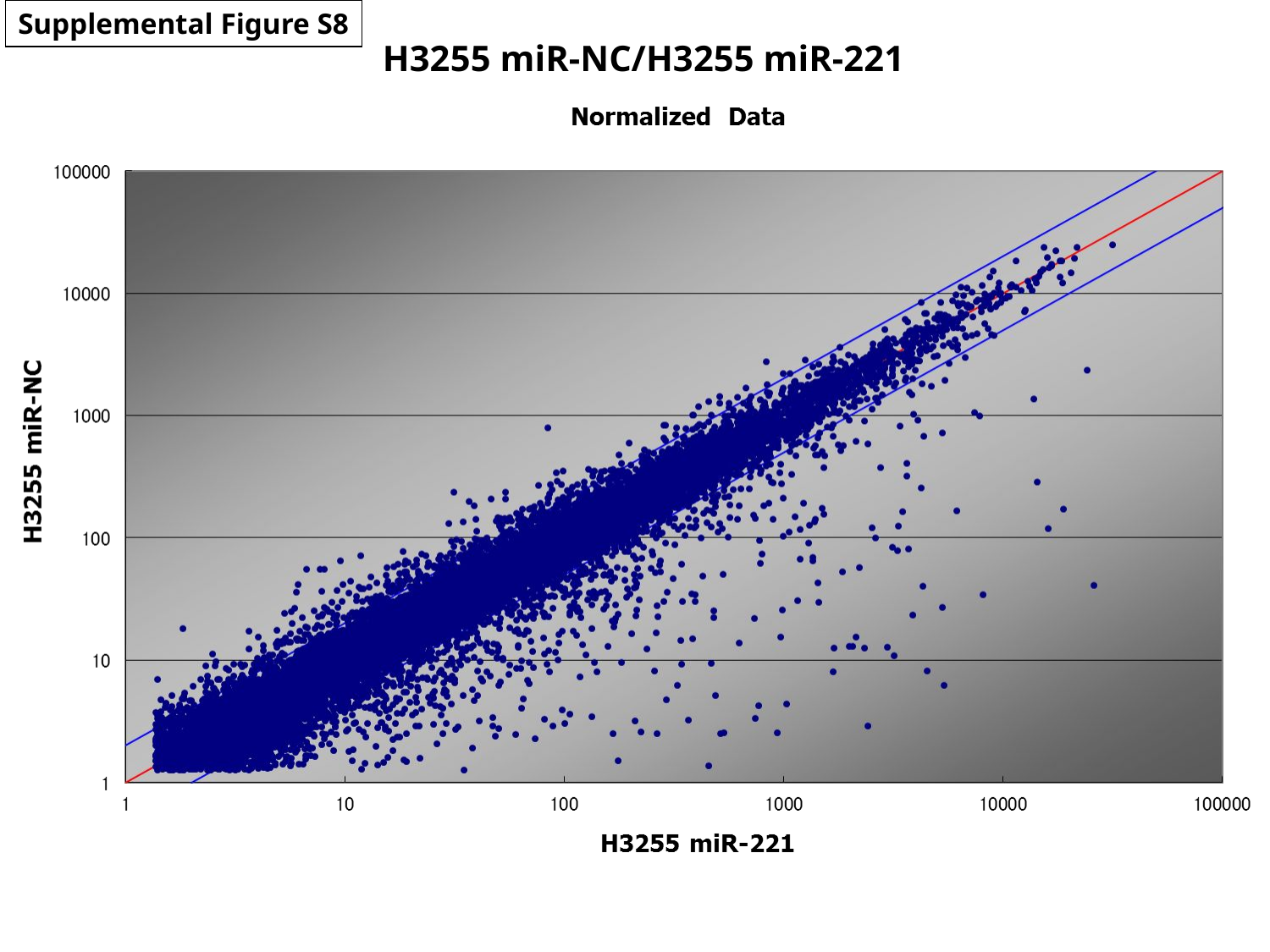

Supplemental Figure S8
# H3255 miR-NC/H3255 miR-221

## Slide 9
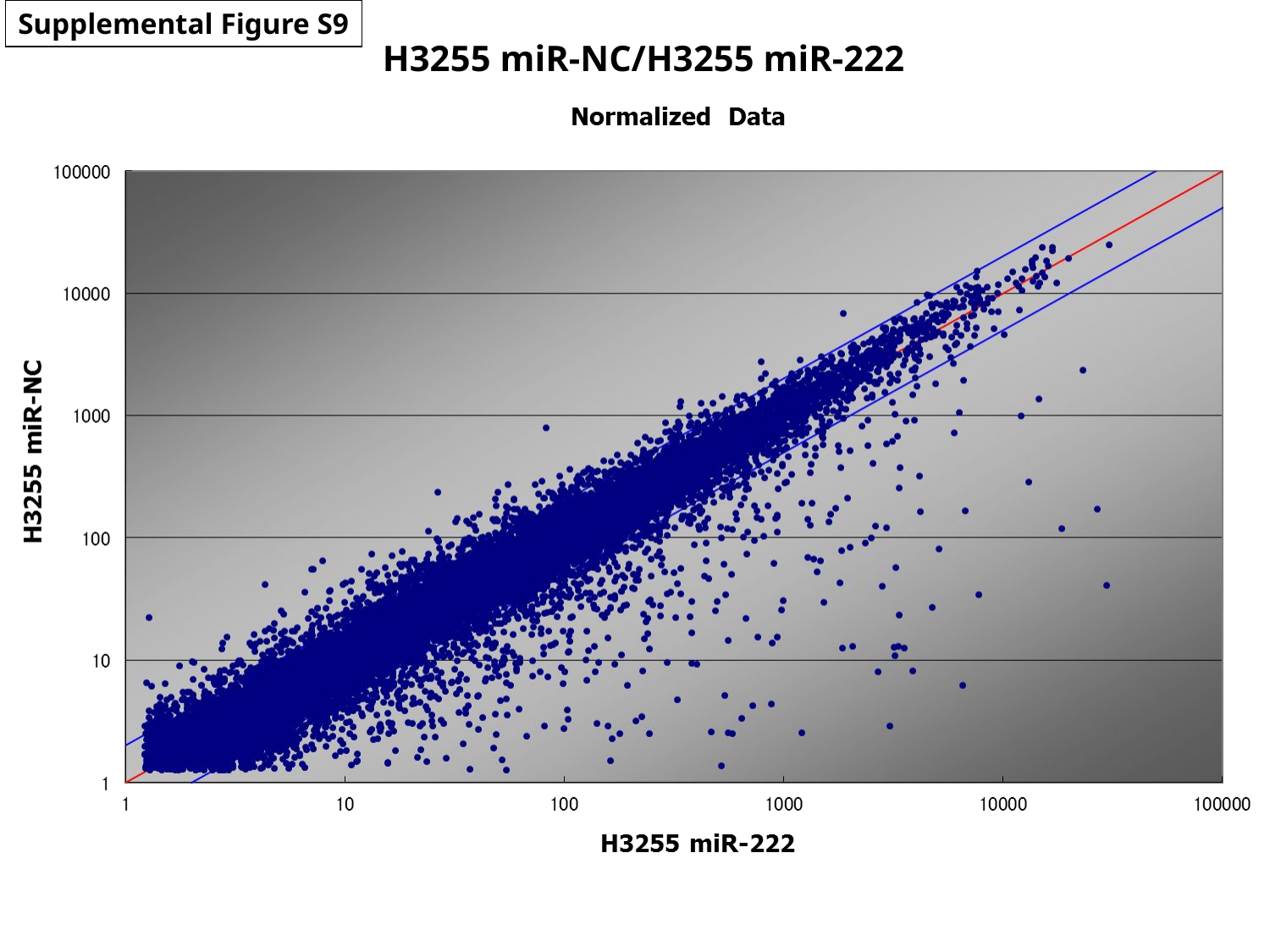

Supplemental Figure S9
# H3255 miR-NC/H3255 miR-222

## Slide 10
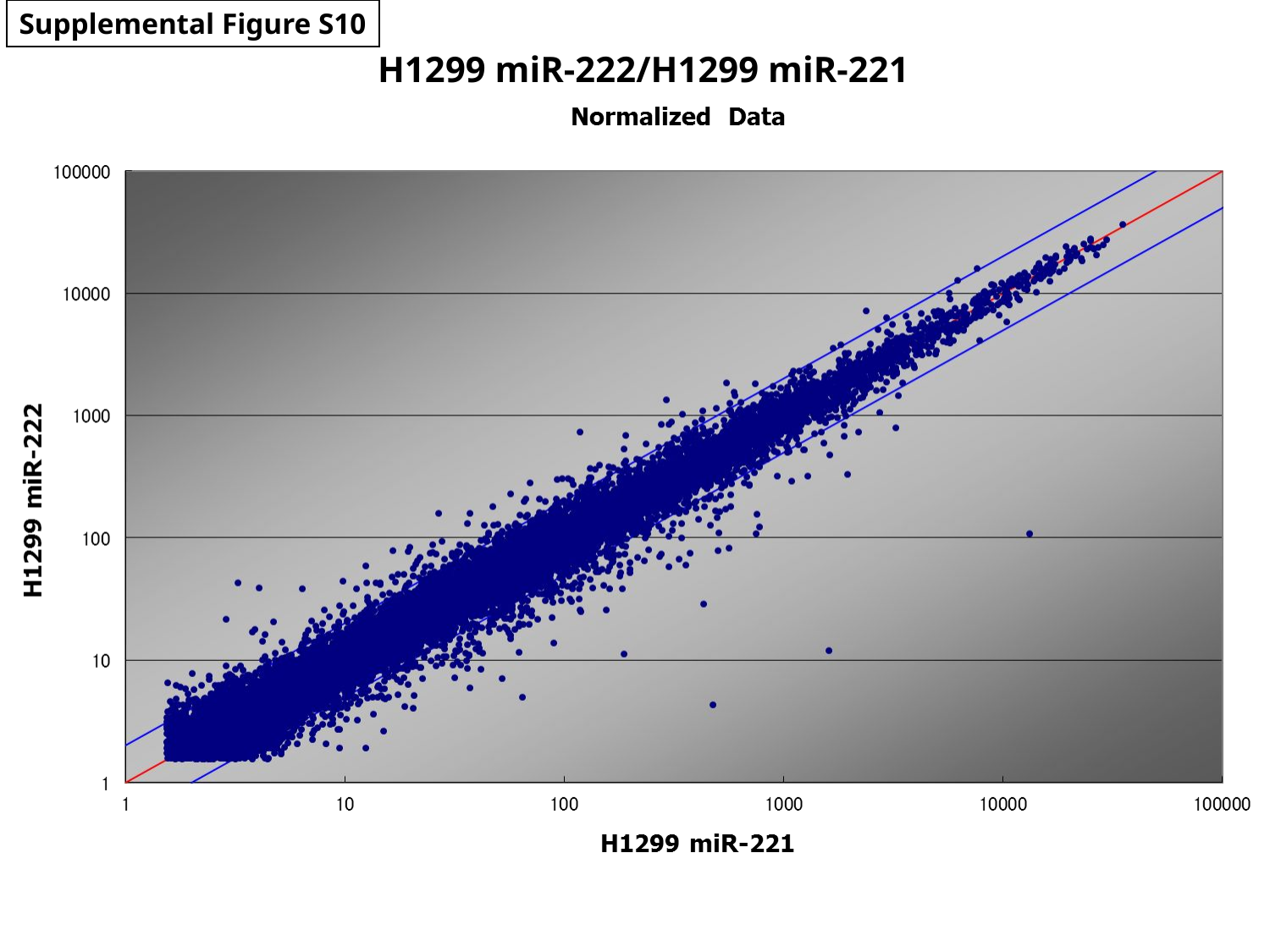

Supplemental Figure S10
# H1299 miR-222/H1299 miR-221

## Slide 11
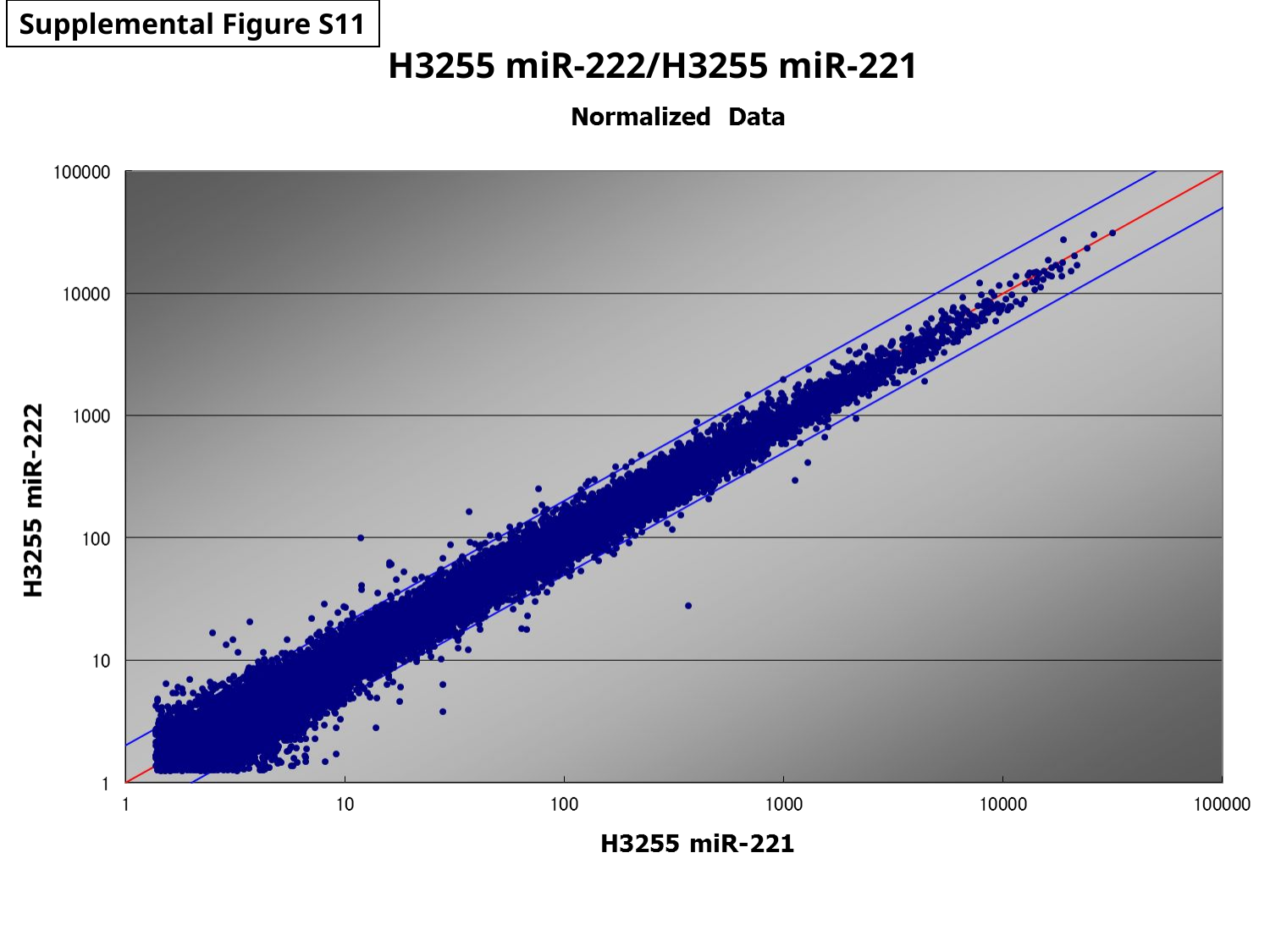

Supplemental Figure S11
# H3255 miR-222/H3255 miR-221

## Slide 12
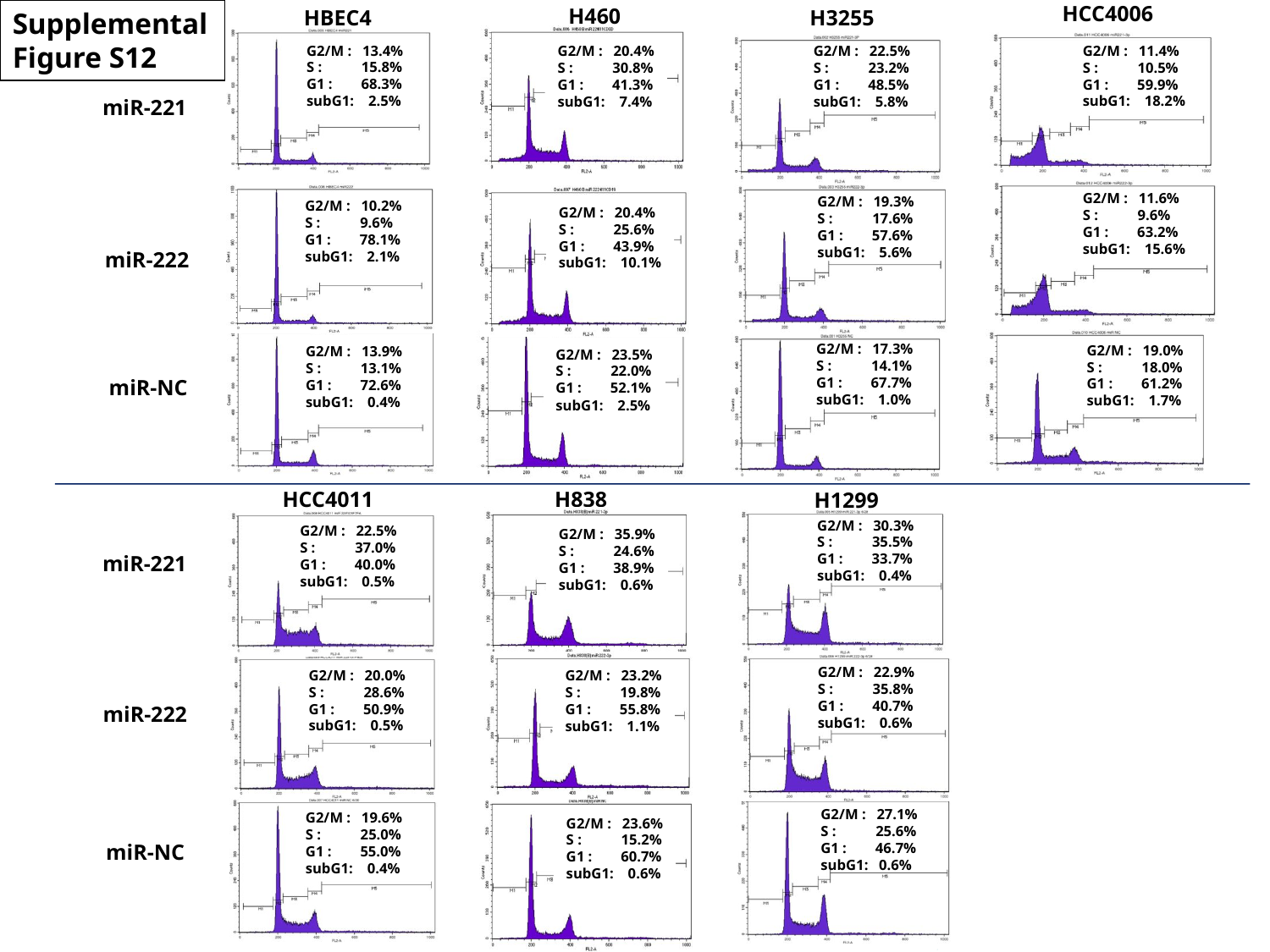

Supplemental
Figure S12
HCC4006
H460
H3255
HBEC4
G2/M : 13.4%
S : 15.8%
G1 : 68.3%
subG1: 2.5%
G2/M : 11.4%
S : 10.5%
G1 : 59.9%
subG1: 18.2%
G2/M : 20.4%
S : 30.8%
G1 : 41.3%
subG1: 7.4%
G2/M : 22.5%
S : 23.2%
G1 : 48.5%
subG1: 5.8%
G2/M : 11.6%
S : 9.6%
G1 : 63.2%
subG1: 15.6%
G2/M : 19.3%
S : 17.6%
G1 : 57.6%
subG1: 5.6%
G2/M : 10.2%
S : 9.6%
G1 : 78.1%
subG1: 2.1%
G2/M : 20.4%
S : 25.6%
G1 : 43.9%
subG1: 10.1%
miR-221
miR-222
G2/M : 17.3%
S : 14.1%
G1 : 67.7%
subG1: 1.0%
G2/M : 19.0%
S : 18.0%
G1 : 61.2%
subG1: 1.7%
G2/M : 13.9%
S : 13.1%
G1 : 72.6%
subG1: 0.4%
G2/M : 23.5%
S : 22.0%
G1 : 52.1%
subG1: 2.5%
miR-NC
H838
HCC4011
H1299
G2/M : 30.3%
S : 35.5%
G1 : 33.7%
subG1: 0.4%
G2/M : 22.5%
S : 37.0%
G1 : 40.0%
subG1: 0.5%
G2/M : 35.9%
S : 24.6%
G1 : 38.9%
subG1: 0.6%
G2/M : 22.9%
S : 35.8%
G1 : 40.7%
subG1: 0.6%
G2/M : 20.0%
S : 28.6%
G1 : 50.9%
subG1: 0.5%
G2/M : 23.2%
S : 19.8%
G1 : 55.8%
subG1: 1.1%
miR-221
miR-222
G2/M : 27.1%
S : 25.6%
G1 : 46.7%
subG1: 0.6%
G2/M : 19.6%
S : 25.0%
G1 : 55.0%
subG1: 0.4%
G2/M : 23.6%
S : 15.2%
G1 : 60.7%
subG1: 0.6%
miR-NC

## Slide 13
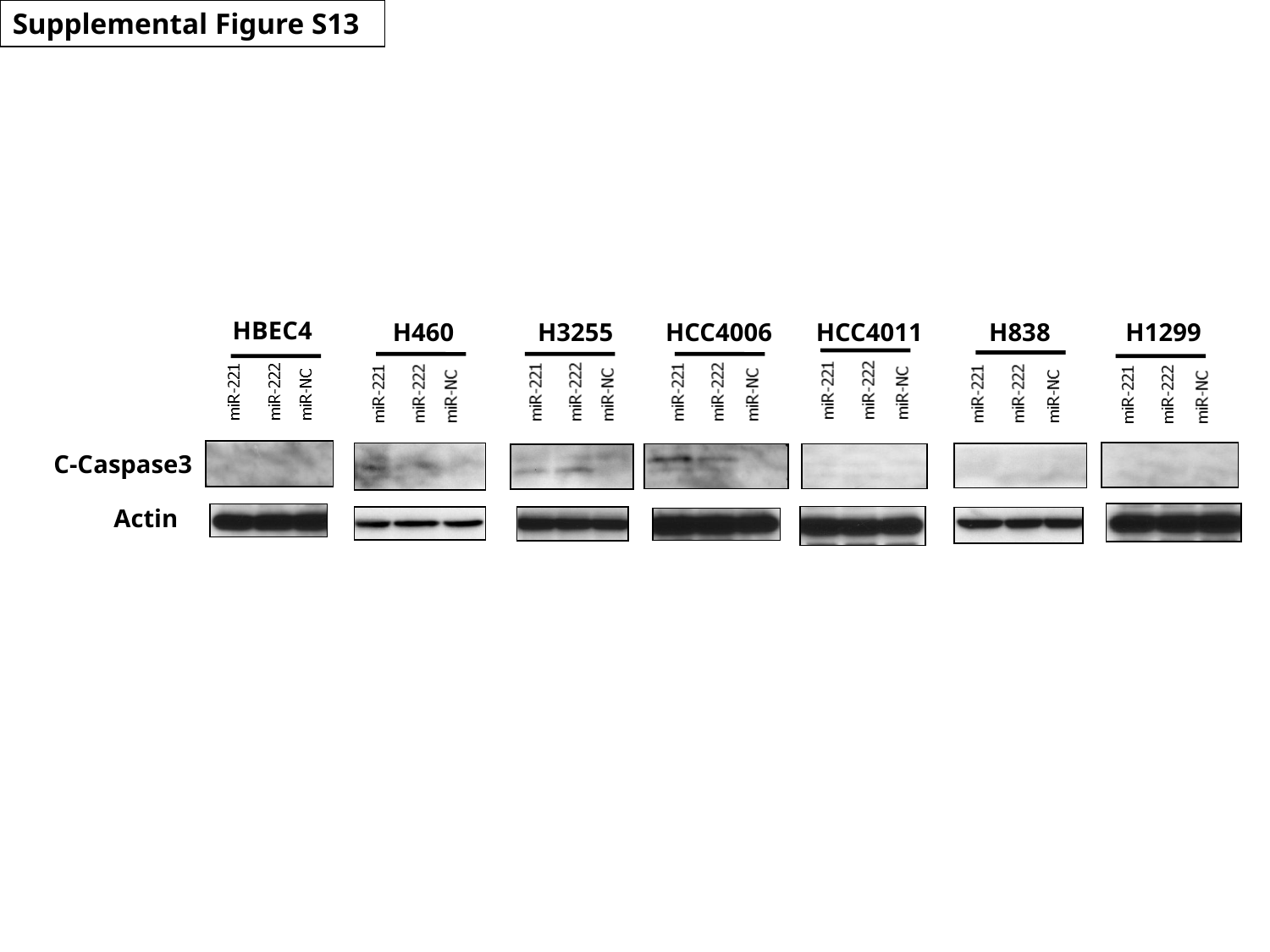

Supplemental Figure S13
H3255
HCC4006
HCC4011
H838
H1299
C-Caspase3
Actin
HBEC4
H460

## Slide 14
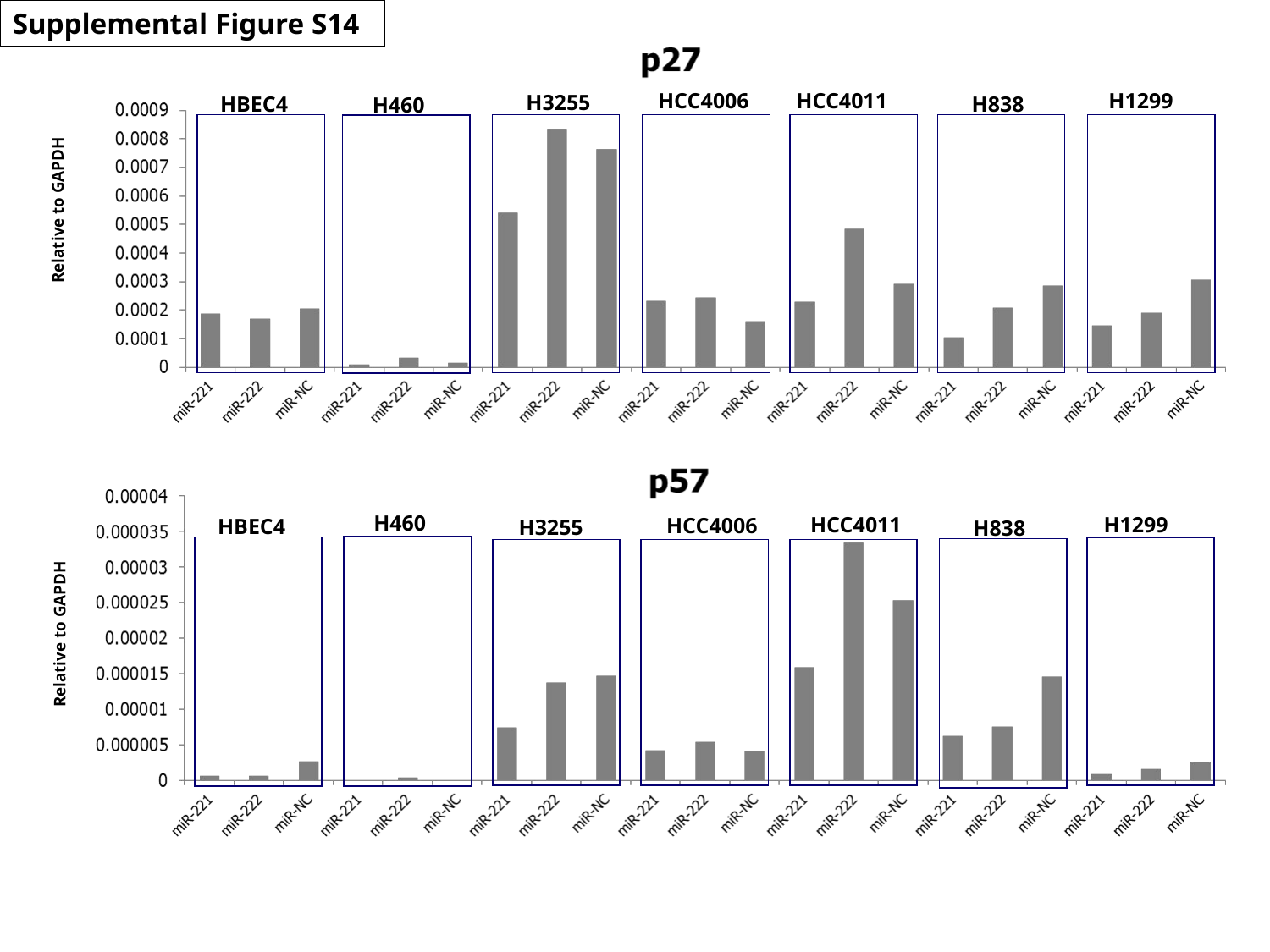

Supplemental Figure S14
HCC4011
H1299
HCC4006
H3255
HBEC4
H838
H460
Relative to GAPDH
H460
HCC4011
H1299
HCC4006
HBEC4
H3255
H838
Relative to GAPDH

## Slide 15
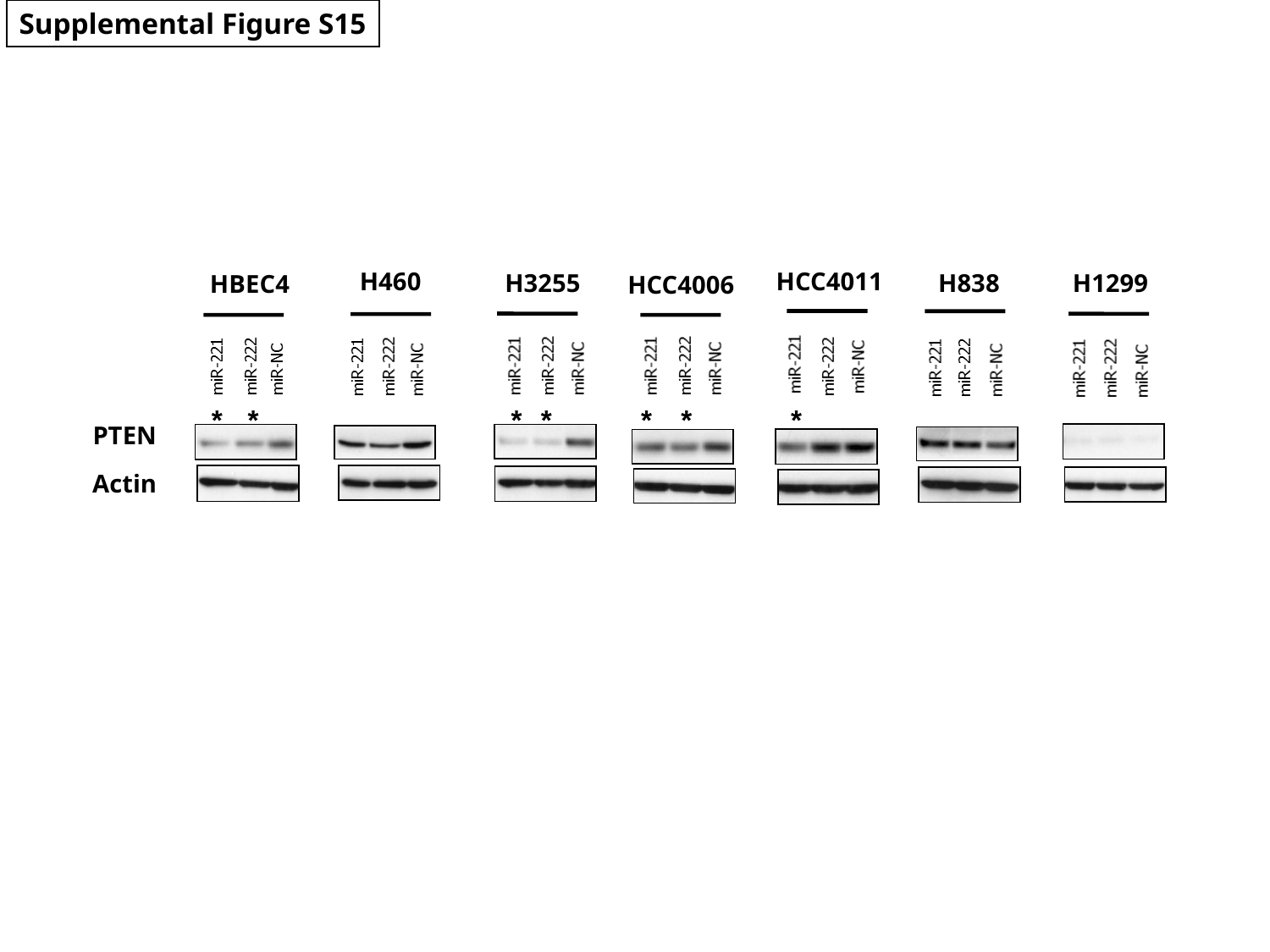

Supplemental Figure S15
H460
HCC4011
H1299
H3255
H838
HBEC4
HCC4006
*
*
*
*
*
*
*
PTEN
Actin

## Slide 16
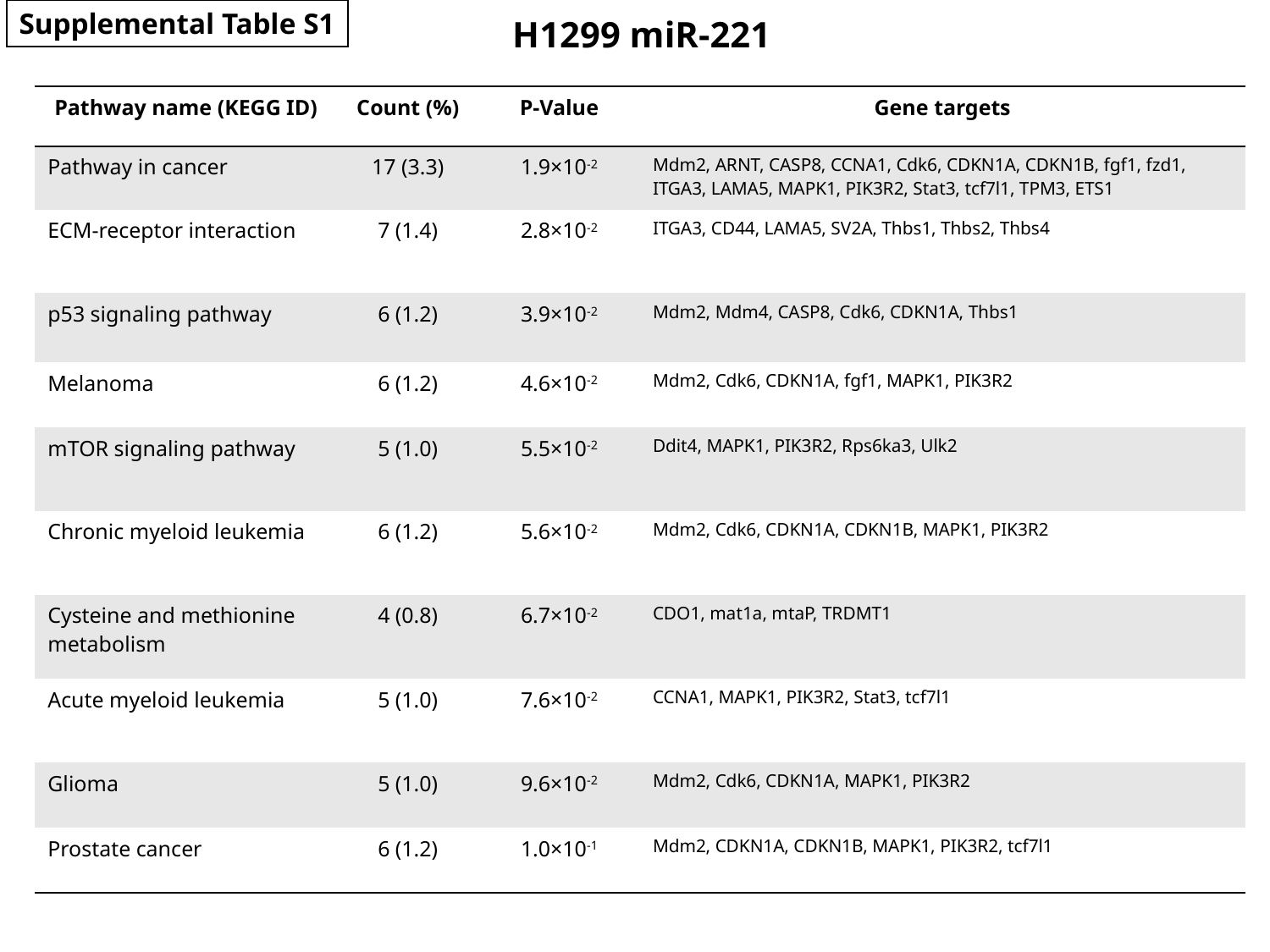

Supplemental Table S1
H1299 miR-221
| Pathway name (KEGG ID) | Count (%) | P-Value | Gene targets |
| --- | --- | --- | --- |
| Pathway in cancer | 17 (3.3) | 1.9×10^-2^ | Mdm2, ARNT, CASP8, CCNA1, Cdk6, CDKN1A, CDKN1B, fgf1, fzd1, ITGA3, LAMA5, MAPK1, PIK3R2, Stat3, tcf7l1, TPM3, ETS1 |
| ECM-receptor interaction | 7 (1.4) | 2.8×10^-2^ | ITGA3, CD44, LAMA5, SV2A, Thbs1, Thbs2, Thbs4 |
| p53 signaling pathway | 6 (1.2) | 3.9×10^-2^ | Mdm2, Mdm4, CASP8, Cdk6, CDKN1A, Thbs1 |
| Melanoma | 6 (1.2) | 4.6×10^-2^ | Mdm2, Cdk6, CDKN1A, fgf1, MAPK1, PIK3R2 |
| mTOR signaling pathway | 5 (1.0) | 5.5×10^-2^ | Ddit4, MAPK1, PIK3R2, Rps6ka3, Ulk2 |
| Chronic myeloid leukemia | 6 (1.2) | 5.6×10^-2^ | Mdm2, Cdk6, CDKN1A, CDKN1B, MAPK1, PIK3R2 |
| Cysteine and methionine metabolism | 4 (0.8) | 6.7×10^-2^ | CDO1, mat1a, mtaP, TRDMT1 |
| Acute myeloid leukemia | 5 (1.0) | 7.6×10^-2^ | CCNA1, MAPK1, PIK3R2, Stat3, tcf7l1 |
| Glioma | 5 (1.0) | 9.6×10^-2^ | Mdm2, Cdk6, CDKN1A, MAPK1, PIK3R2 |
| Prostate cancer | 6 (1.2) | 1.0×10^-1^ | Mdm2, CDKN1A, CDKN1B, MAPK1, PIK3R2, tcf7l1 |

## Slide 17
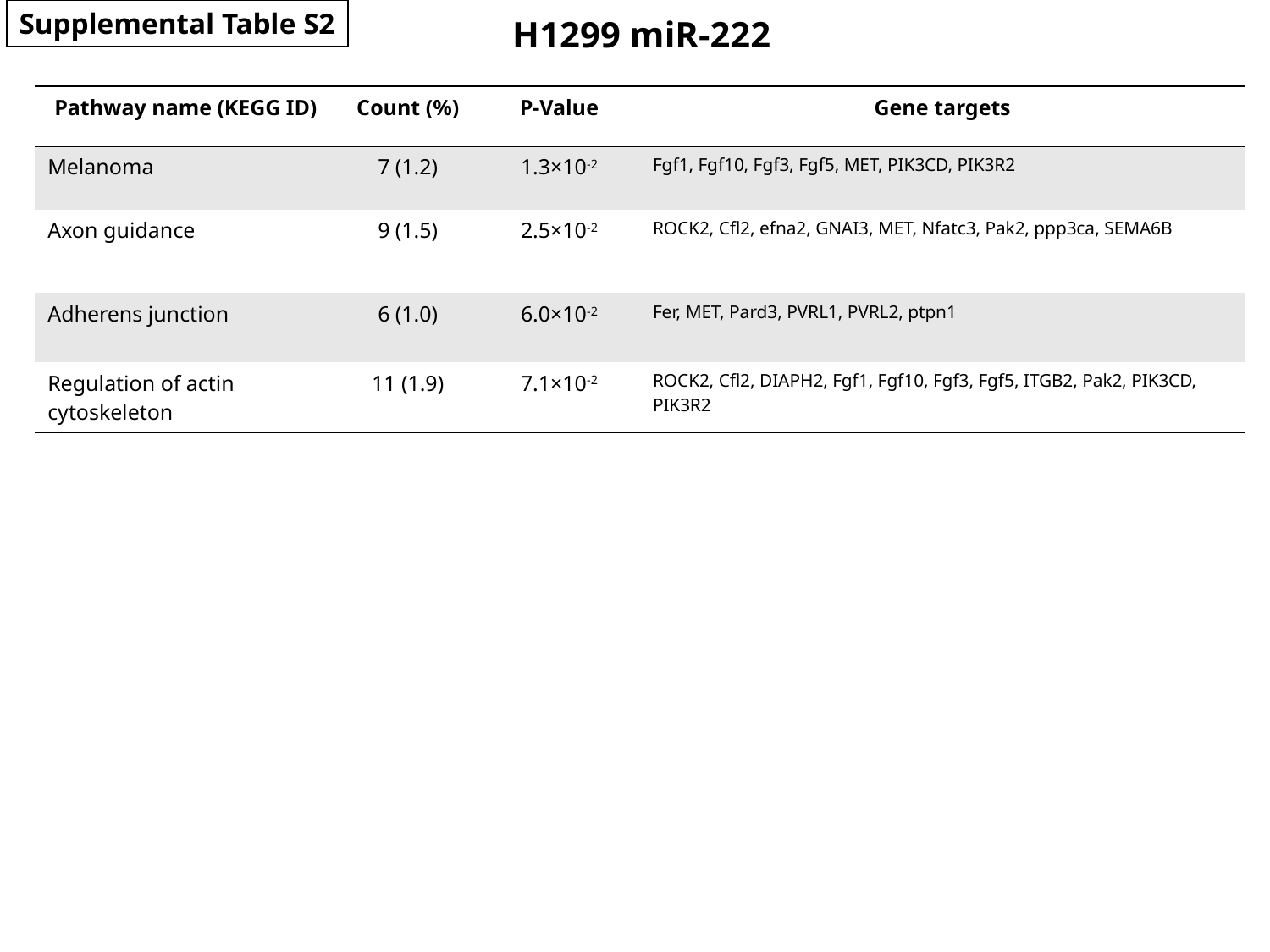

Supplemental Table S2
H1299 miR-222
| Pathway name (KEGG ID) | Count (%) | P-Value | Gene targets |
| --- | --- | --- | --- |
| Melanoma | 7 (1.2) | 1.3×10^-2^ | Fgf1, Fgf10, Fgf3, Fgf5, MET, PIK3CD, PIK3R2 |
| Axon guidance | 9 (1.5) | 2.5×10^-2^ | ROCK2, Cfl2, efna2, GNAI3, MET, Nfatc3, Pak2, ppp3ca, SEMA6B |
| Adherens junction | 6 (1.0) | 6.0×10^-2^ | Fer, MET, Pard3, PVRL1, PVRL2, ptpn1 |
| Regulation of actin cytoskeleton | 11 (1.9) | 7.1×10^-2^ | ROCK2, Cfl2, DIAPH2, Fgf1, Fgf10, Fgf3, Fgf5, ITGB2, Pak2, PIK3CD, PIK3R2 |

## Slide 18
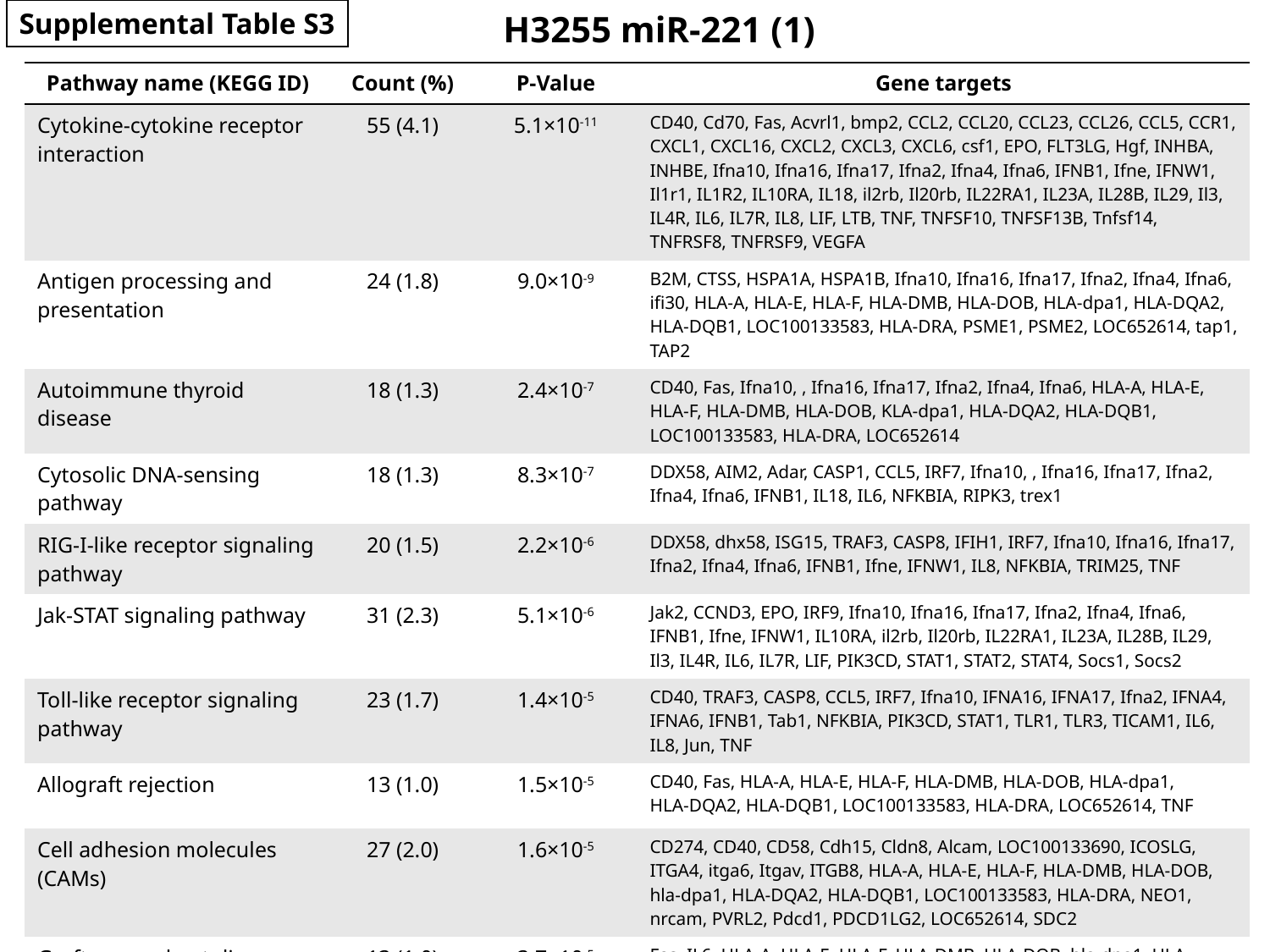

Supplemental Table S3
H3255 miR-221 (1)
| Pathway name (KEGG ID) | Count (%) | P-Value | Gene targets |
| --- | --- | --- | --- |
| Cytokine-cytokine receptor interaction | 55 (4.1) | 5.1×10^-11^ | CD40, Cd70, Fas, Acvrl1, bmp2, CCL2, CCL20, CCL23, CCL26, CCL5, CCR1, CXCL1, CXCL16, CXCL2, CXCL3, CXCL6, csf1, EPO, FLT3LG, Hgf, INHBA, INHBE, Ifna10, Ifna16, Ifna17, Ifna2, Ifna4, Ifna6, IFNB1, Ifne, IFNW1, Il1r1, IL1R2, IL10RA, IL18, il2rb, Il20rb, IL22RA1, IL23A, IL28B, IL29, Il3, IL4R, IL6, IL7R, IL8, LIF, LTB, TNF, TNFSF10, TNFSF13B, Tnfsf14, TNFRSF8, TNFRSF9, VEGFA |
| Antigen processing and presentation | 24 (1.8) | 9.0×10^-9^ | B2M, CTSS, HSPA1A, HSPA1B, Ifna10, Ifna16, Ifna17, Ifna2, Ifna4, Ifna6, ifi30, HLA-A, HLA-E, HLA-F, HLA-DMB, HLA-DOB, HLA-dpa1, HLA-DQA2, HLA-DQB1, LOC100133583, HLA-DRA, PSME1, PSME2, LOC652614, tap1, TAP2 |
| Autoimmune thyroid disease | 18 (1.3) | 2.4×10^-7^ | CD40, Fas, Ifna10, , Ifna16, Ifna17, Ifna2, Ifna4, Ifna6, HLA-A, HLA-E, HLA-F, HLA-DMB, HLA-DOB, KLA-dpa1, HLA-DQA2, HLA-DQB1, LOC100133583, HLA-DRA, LOC652614 |
| Cytosolic DNA-sensing pathway | 18 (1.3) | 8.3×10^-7^ | DDX58, AIM2, Adar, CASP1, CCL5, IRF7, Ifna10, , Ifna16, Ifna17, Ifna2, Ifna4, Ifna6, IFNB1, IL18, IL6, NFKBIA, RIPK3, trex1 |
| RIG-I-like receptor signaling pathway | 20 (1.5) | 2.2×10^-6^ | DDX58, dhx58, ISG15, TRAF3, CASP8, IFIH1, IRF7, Ifna10, Ifna16, Ifna17, Ifna2, Ifna4, Ifna6, IFNB1, Ifne, IFNW1, IL8, NFKBIA, TRIM25, TNF |
| Jak-STAT signaling pathway | 31 (2.3) | 5.1×10^-6^ | Jak2, CCND3, EPO, IRF9, Ifna10, Ifna16, Ifna17, Ifna2, Ifna4, Ifna6, IFNB1, Ifne, IFNW1, IL10RA, il2rb, Il20rb, IL22RA1, IL23A, IL28B, IL29, Il3, IL4R, IL6, IL7R, LIF, PIK3CD, STAT1, STAT2, STAT4, Socs1, Socs2 |
| Toll-like receptor signaling pathway | 23 (1.7) | 1.4×10^-5^ | CD40, TRAF3, CASP8, CCL5, IRF7, Ifna10, IFNA16, IFNA17, Ifna2, IFNA4, IFNA6, IFNB1, Tab1, NFKBIA, PIK3CD, STAT1, TLR1, TLR3, TICAM1, IL6, IL8, Jun, TNF |
| Allograft rejection | 13 (1.0) | 1.5×10^-5^ | CD40, Fas, HLA-A, HLA-E, HLA-F, HLA-DMB, HLA-DOB, HLA-dpa1, HLA-DQA2, HLA-DQB1, LOC100133583, HLA-DRA, LOC652614, TNF |
| Cell adhesion molecules (CAMs) | 27 (2.0) | 1.6×10^-5^ | CD274, CD40, CD58, Cdh15, Cldn8, Alcam, LOC100133690, ICOSLG, ITGA4, itga6, Itgav, ITGB8, HLA-A, HLA-E, HLA-F, HLA-DMB, HLA-DOB, hla-dpa1, HLA-DQA2, HLA-DQB1, LOC100133583, HLA-DRA, NEO1, nrcam, PVRL2, Pdcd1, PDCD1LG2, LOC652614, SDC2 |
| Graft-versus-host disease | 13 (1.0) | 3.7×10^-5^ | Fas, IL6, HLA-A, HLA-E, HLA-F, HLA-DMB, HLA-DOB, hla-dpa1, HLA-DQA2, HLA-DQB1, LOC100133583, HLA-DRA, LOC652614, TNF |

## Slide 19
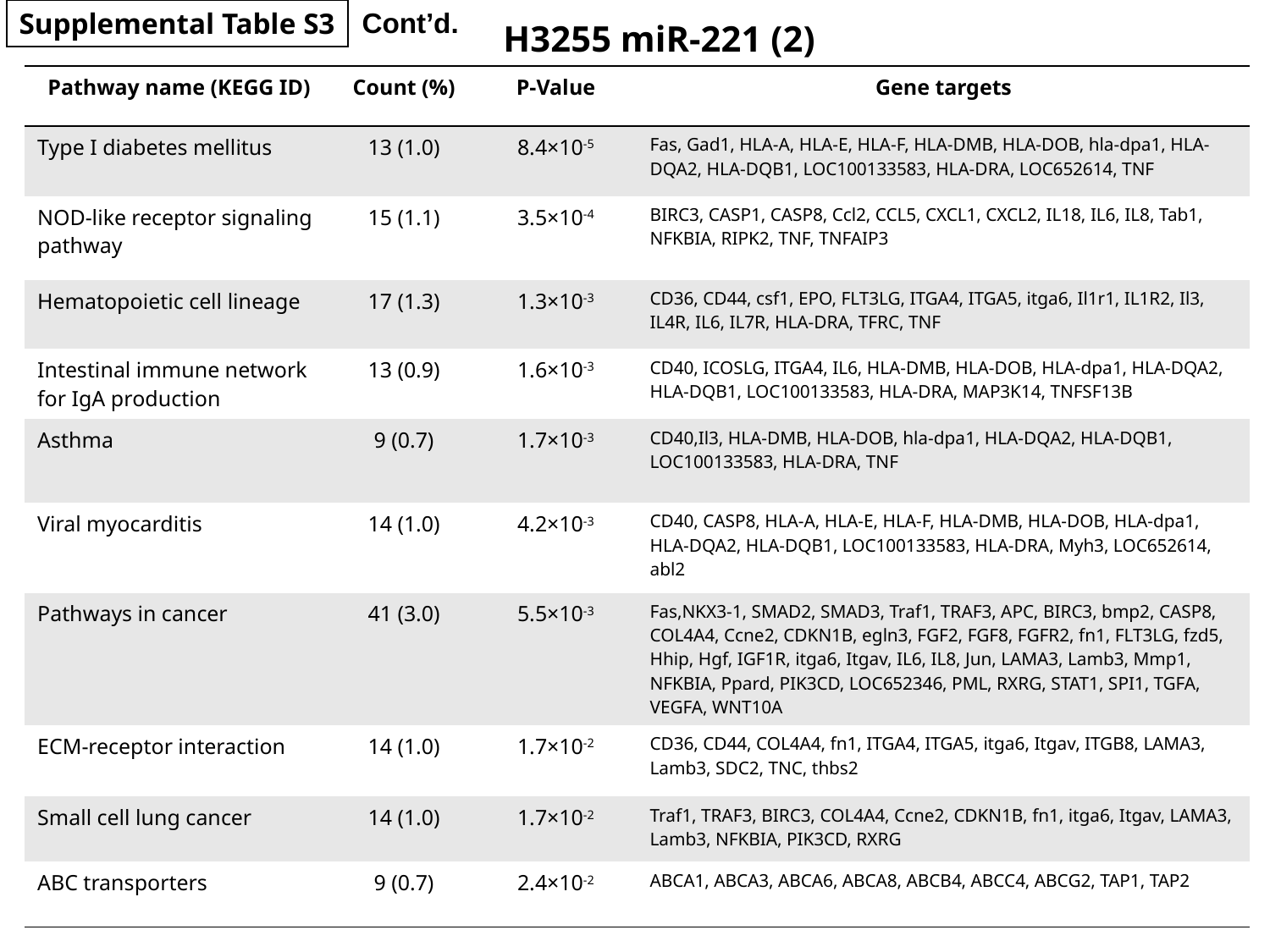

Supplemental Table S3
Cont’d.
H3255 miR-221 (2)
| Pathway name (KEGG ID) | Count (%) | P-Value | Gene targets |
| --- | --- | --- | --- |
| Type I diabetes mellitus | 13 (1.0) | 8.4×10^-5^ | Fas, Gad1, HLA-A, HLA-E, HLA-F, HLA-DMB, HLA-DOB, hla-dpa1, HLA-DQA2, HLA-DQB1, LOC100133583, HLA-DRA, LOC652614, TNF |
| NOD-like receptor signaling pathway | 15 (1.1) | 3.5×10^-4^ | BIRC3, CASP1, CASP8, Ccl2, CCL5, CXCL1, CXCL2, IL18, IL6, IL8, Tab1, NFKBIA, RIPK2, TNF, TNFAIP3 |
| Hematopoietic cell lineage | 17 (1.3) | 1.3×10^-3^ | CD36, CD44, csf1, EPO, FLT3LG, ITGA4, ITGA5, itga6, Il1r1, IL1R2, Il3, IL4R, IL6, IL7R, HLA-DRA, TFRC, TNF |
| Intestinal immune network for IgA production | 13 (0.9) | 1.6×10^-3^ | CD40, ICOSLG, ITGA4, IL6, HLA-DMB, HLA-DOB, HLA-dpa1, HLA-DQA2, HLA-DQB1, LOC100133583, HLA-DRA, MAP3K14, TNFSF13B |
| Asthma | 9 (0.7) | 1.7×10^-3^ | CD40,Il3, HLA-DMB, HLA-DOB, hla-dpa1, HLA-DQA2, HLA-DQB1, LOC100133583, HLA-DRA, TNF |
| Viral myocarditis | 14 (1.0) | 4.2×10^-3^ | CD40, CASP8, HLA-A, HLA-E, HLA-F, HLA-DMB, HLA-DOB, HLA-dpa1, HLA-DQA2, HLA-DQB1, LOC100133583, HLA-DRA, Myh3, LOC652614, abl2 |
| Pathways in cancer | 41 (3.0) | 5.5×10^-3^ | Fas,NKX3-1, SMAD2, SMAD3, Traf1, TRAF3, APC, BIRC3, bmp2, CASP8, COL4A4, Ccne2, CDKN1B, egln3, FGF2, FGF8, FGFR2, fn1, FLT3LG, fzd5, Hhip, Hgf, IGF1R, itga6, Itgav, IL6, IL8, Jun, LAMA3, Lamb3, Mmp1, NFKBIA, Ppard, PIK3CD, LOC652346, PML, RXRG, STAT1, SPI1, TGFA, VEGFA, WNT10A |
| ECM-receptor interaction | 14 (1.0) | 1.7×10^-2^ | CD36, CD44, COL4A4, fn1, ITGA4, ITGA5, itga6, Itgav, ITGB8, LAMA3, Lamb3, SDC2, TNC, thbs2 |
| Small cell lung cancer | 14 (1.0) | 1.7×10^-2^ | Traf1, TRAF3, BIRC3, COL4A4, Ccne2, CDKN1B, fn1, itga6, Itgav, LAMA3, Lamb3, NFKBIA, PIK3CD, RXRG |
| ABC transporters | 9 (0.7) | 2.4×10^-2^ | ABCA1, ABCA3, ABCA6, ABCA8, ABCB4, ABCC4, ABCG2, TAP1, TAP2 |

## Slide 20
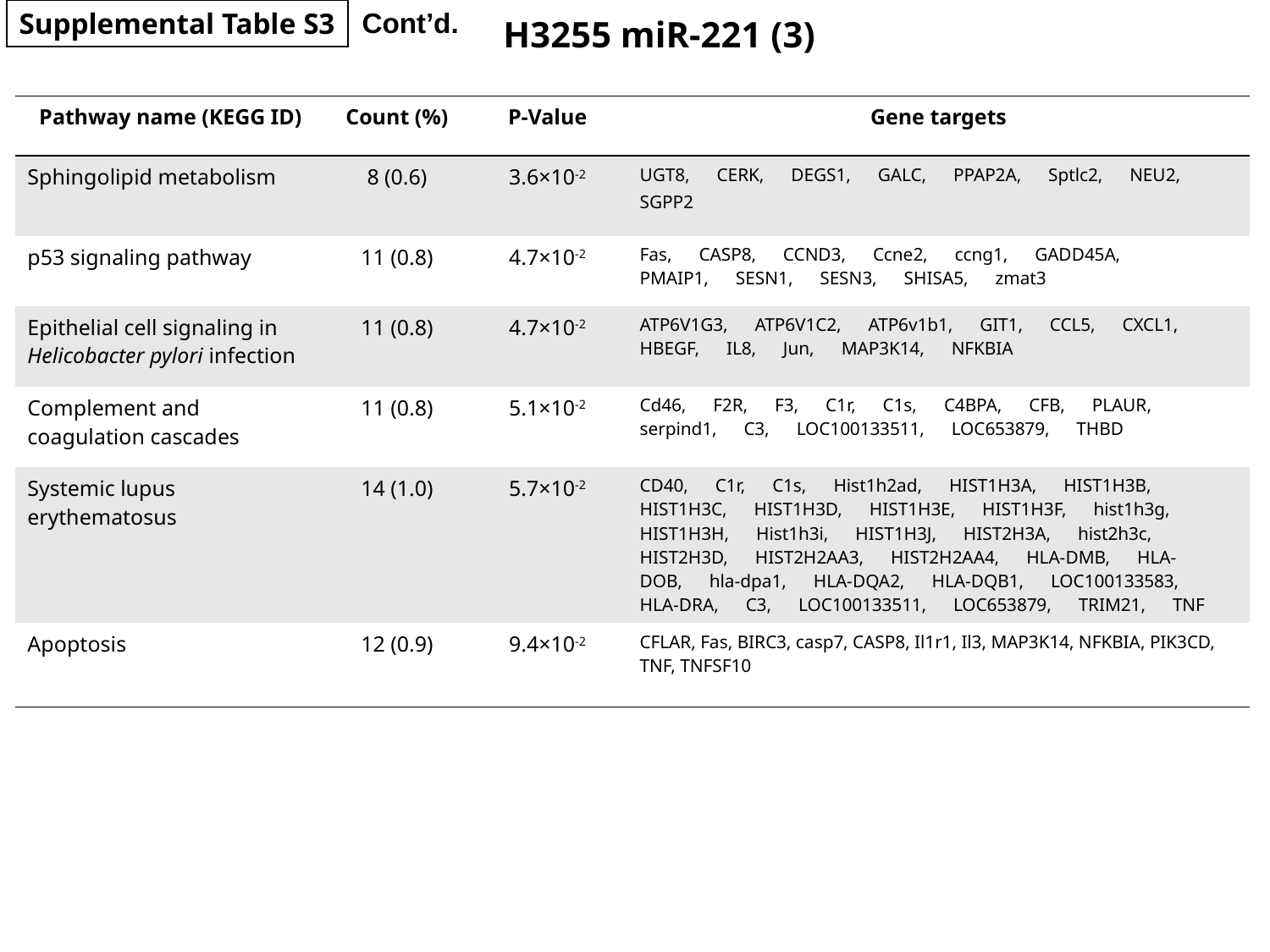

Supplemental Table S3
Cont’d.
H3255 miR-221 (3)
| Pathway name (KEGG ID) | Count (%) | P-Value | Gene targets |
| --- | --- | --- | --- |
| Sphingolipid metabolism | 8 (0.6) | 3.6×10^-2^ | UGT8,　CERK,　DEGS1,　GALC,　PPAP2A,　Sptlc2,　NEU2,　SGPP2 |
| p53 signaling pathway | 11 (0.8) | 4.7×10^-2^ | Fas,　CASP8,　CCND3,　Ccne2,　ccng1,　GADD45A,　PMAIP1,　SESN1,　SESN3,　SHISA5,　zmat3 |
| Epithelial cell signaling in Helicobacter pylori infection | 11 (0.8) | 4.7×10^-2^ | ATP6V1G3,　ATP6V1C2,　ATP6v1b1,　GIT1,　CCL5,　CXCL1,　HBEGF,　IL8,　Jun,　MAP3K14,　NFKBIA |
| Complement and coagulation cascades | 11 (0.8) | 5.1×10^-2^ | Cd46,　F2R,　F3,　C1r,　C1s,　C4BPA,　CFB,　PLAUR,　serpind1,　C3,　LOC100133511,　LOC653879,　THBD |
| Systemic lupus erythematosus | 14 (1.0) | 5.7×10^-2^ | CD40,　C1r,　C1s,　Hist1h2ad,　HIST1H3A,　HIST1H3B,　HIST1H3C,　HIST1H3D,　HIST1H3E,　HIST1H3F,　hist1h3g,　HIST1H3H,　Hist1h3i,　HIST1H3J,　HIST2H3A,　hist2h3c,　HIST2H3D,　HIST2H2AA3,　HIST2H2AA4,　HLA-DMB,　HLA-DOB,　hla-dpa1,　HLA-DQA2,　HLA-DQB1,　LOC100133583,　HLA-DRA,　C3,　LOC100133511,　LOC653879,　TRIM21,　TNF |
| Apoptosis | 12 (0.9) | 9.4×10^-2^ | CFLAR, Fas, BIRC3, casp7, CASP8, Il1r1, Il3, MAP3K14, NFKBIA, PIK3CD,　TNF, TNFSF10 |

## Slide 21
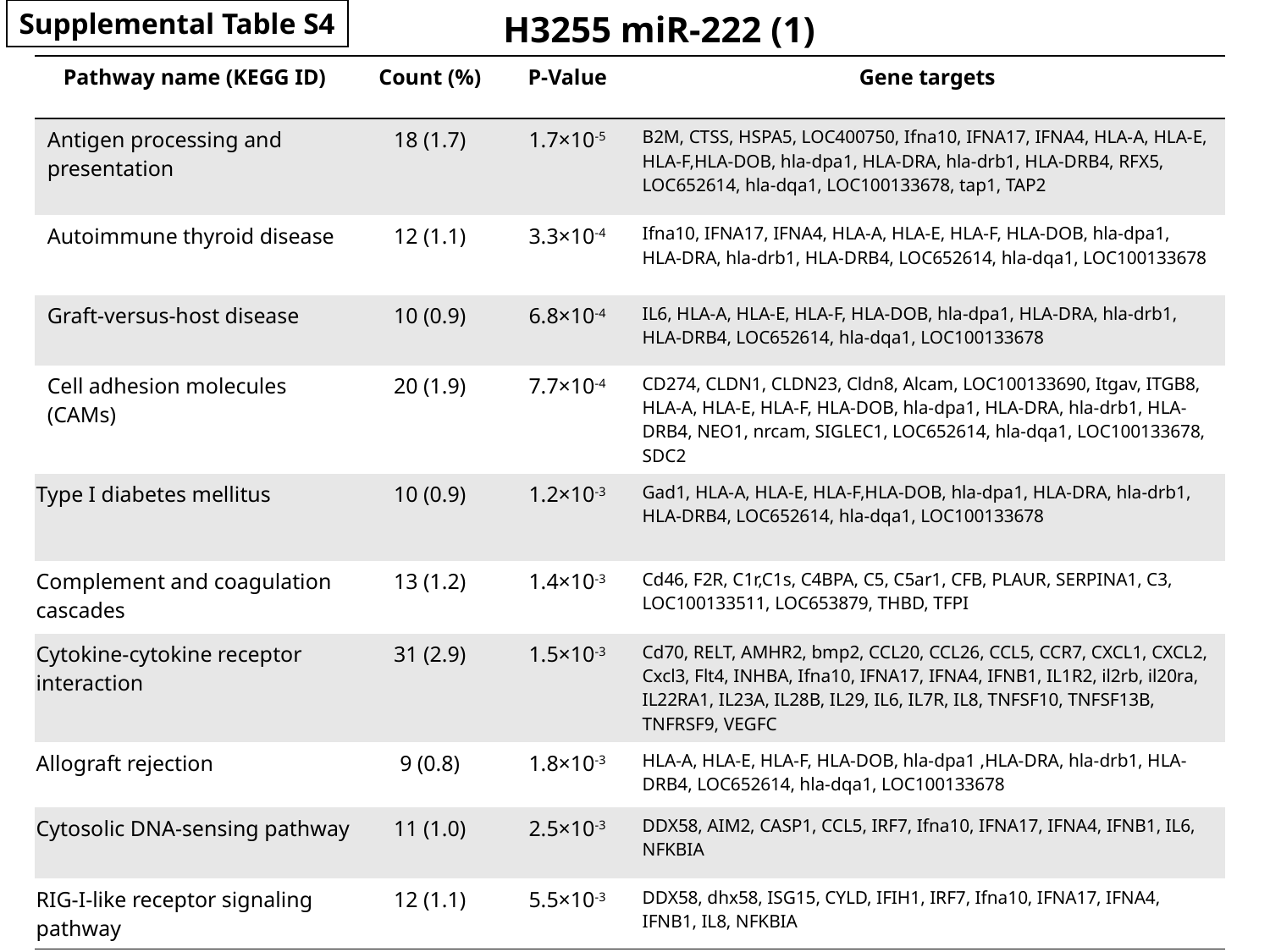

Supplemental Table S4
H3255 miR-222 (1)
| Pathway name (KEGG ID) | Count (%) | P-Value | Gene targets |
| --- | --- | --- | --- |
| Antigen processing and presentation | 18 (1.7) | 1.7×10^-5^ | B2M, CTSS, HSPA5, LOC400750, Ifna10, IFNA17, IFNA4, HLA-A, HLA-E, HLA-F,HLA-DOB, hla-dpa1, HLA-DRA, hla-drb1, HLA-DRB4, RFX5, LOC652614, hla-dqa1, LOC100133678, tap1, TAP2 |
| Autoimmune thyroid disease | 12 (1.1) | 3.3×10^-4^ | Ifna10, IFNA17, IFNA4, HLA-A, HLA-E, HLA-F, HLA-DOB, hla-dpa1, HLA-DRA, hla-drb1, HLA-DRB4, LOC652614, hla-dqa1, LOC100133678 |
| Graft-versus-host disease | 10 (0.9) | 6.8×10^-4^ | IL6, HLA-A, HLA-E, HLA-F, HLA-DOB, hla-dpa1, HLA-DRA, hla-drb1, HLA-DRB4, LOC652614, hla-dqa1, LOC100133678 |
| Cell adhesion molecules (CAMs) | 20 (1.9) | 7.7×10^-4^ | CD274, CLDN1, CLDN23, Cldn8, Alcam, LOC100133690, Itgav, ITGB8, HLA-A, HLA-E, HLA-F, HLA-DOB, hla-dpa1, HLA-DRA, hla-drb1, HLA-DRB4, NEO1, nrcam, SIGLEC1, LOC652614, hla-dqa1, LOC100133678, SDC2 |
| Type I diabetes mellitus | 10 (0.9) | 1.2×10^-3^ | Gad1, HLA-A, HLA-E, HLA-F,HLA-DOB, hla-dpa1, HLA-DRA, hla-drb1, HLA-DRB4, LOC652614, hla-dqa1, LOC100133678 |
| Complement and coagulation cascades | 13 (1.2) | 1.4×10^-3^ | Cd46, F2R, C1r,C1s, C4BPA, C5, C5ar1, CFB, PLAUR, SERPINA1, C3, LOC100133511, LOC653879, THBD, TFPI |
| Cytokine-cytokine receptor interaction | 31 (2.9) | 1.5×10^-3^ | Cd70, RELT, AMHR2, bmp2, CCL20, CCL26, CCL5, CCR7, CXCL1, CXCL2, Cxcl3, Flt4, INHBA, Ifna10, IFNA17, IFNA4, IFNB1, IL1R2, il2rb, il20ra, IL22RA1, IL23A, IL28B, IL29, IL6, IL7R, IL8, TNFSF10, TNFSF13B, TNFRSF9, VEGFC |
| Allograft rejection | 9 (0.8) | 1.8×10^-3^ | HLA-A, HLA-E, HLA-F, HLA-DOB, hla-dpa1 ,HLA-DRA, hla-drb1, HLA-DRB4, LOC652614, hla-dqa1, LOC100133678 |
| Cytosolic DNA-sensing pathway | 11 (1.0) | 2.5×10^-3^ | DDX58, AIM2, CASP1, CCL5, IRF7, Ifna10, IFNA17, IFNA4, IFNB1, IL6, NFKBIA |
| RIG-I-like receptor signaling pathway | 12 (1.1) | 5.5×10^-3^ | DDX58, dhx58, ISG15, CYLD, IFIH1, IRF7, Ifna10, IFNA17, IFNA4, IFNB1, IL8, NFKBIA |

## Slide 22
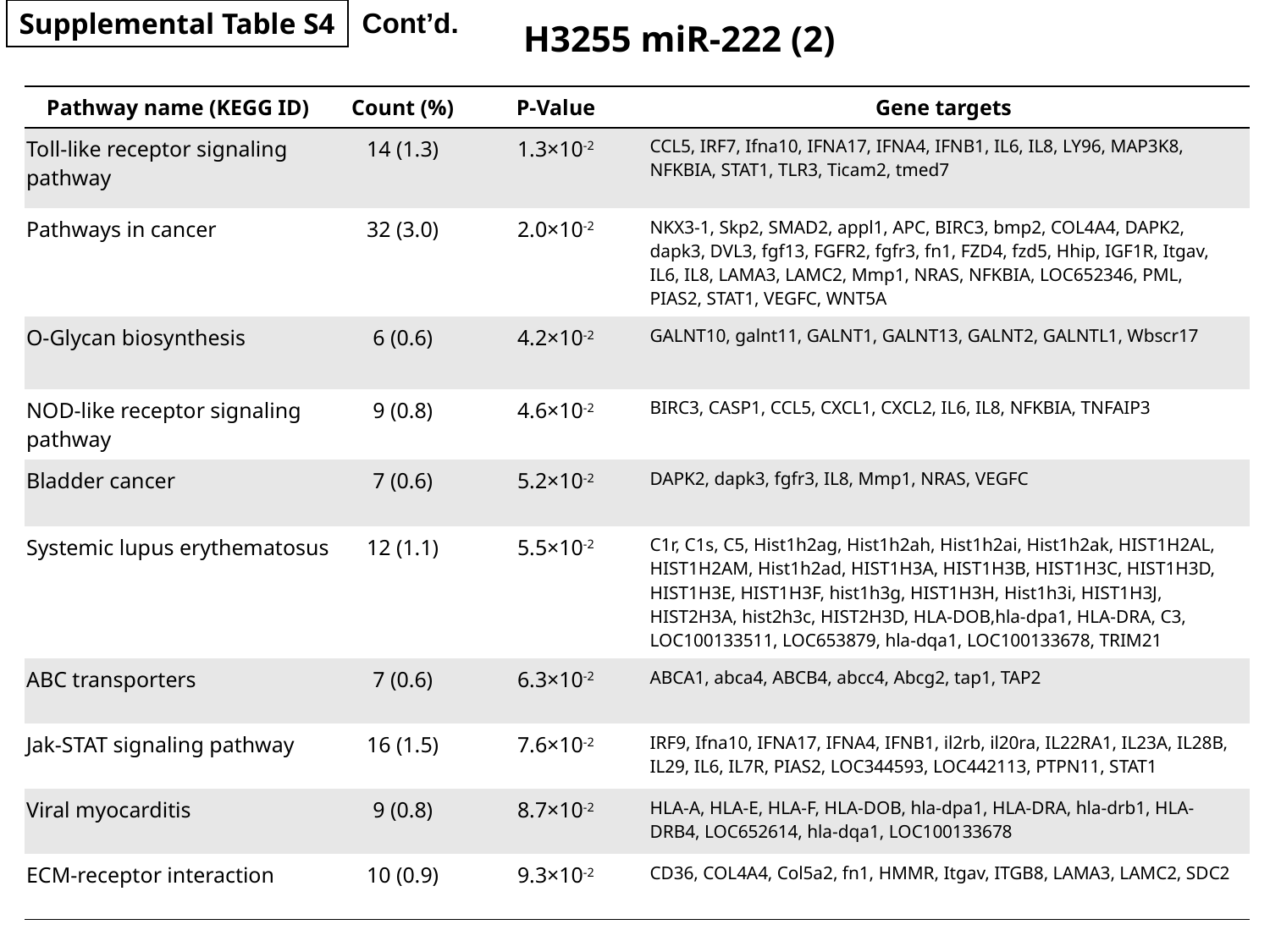

Supplemental Table S4
Cont’d.
H3255 miR-222 (2)
| Pathway name (KEGG ID) | Count (%) | P-Value | Gene targets |
| --- | --- | --- | --- |
| Toll-like receptor signaling pathway | 14 (1.3) | 1.3×10^-2^ | CCL5, IRF7, Ifna10, IFNA17, IFNA4, IFNB1, IL6, IL8, LY96, MAP3K8, NFKBIA, STAT1, TLR3, Ticam2, tmed7 |
| Pathways in cancer | 32 (3.0) | 2.0×10^-2^ | NKX3-1, Skp2, SMAD2, appl1, APC, BIRC3, bmp2, COL4A4, DAPK2, dapk3, DVL3, fgf13, FGFR2, fgfr3, fn1, FZD4, fzd5, Hhip, IGF1R, Itgav, IL6, IL8, LAMA3, LAMC2, Mmp1, NRAS, NFKBIA, LOC652346, PML, PIAS2, STAT1, VEGFC, WNT5A |
| O-Glycan biosynthesis | 6 (0.6) | 4.2×10^-2^ | GALNT10, galnt11, GALNT1, GALNT13, GALNT2, GALNTL1, Wbscr17 |
| NOD-like receptor signaling pathway | 9 (0.8) | 4.6×10^-2^ | BIRC3, CASP1, CCL5, CXCL1, CXCL2, IL6, IL8, NFKBIA, TNFAIP3 |
| Bladder cancer | 7 (0.6) | 5.2×10^-2^ | DAPK2, dapk3, fgfr3, IL8, Mmp1, NRAS, VEGFC |
| Systemic lupus erythematosus | 12 (1.1) | 5.5×10^-2^ | C1r, C1s, C5, Hist1h2ag, Hist1h2ah, Hist1h2ai, Hist1h2ak, HIST1H2AL, HIST1H2AM, Hist1h2ad, HIST1H3A, HIST1H3B, HIST1H3C, HIST1H3D, HIST1H3E, HIST1H3F, hist1h3g, HIST1H3H, Hist1h3i, HIST1H3J, HIST2H3A, hist2h3c, HIST2H3D, HLA-DOB,hla-dpa1, HLA-DRA, C3, LOC100133511, LOC653879, hla-dqa1, LOC100133678, TRIM21 |
| ABC transporters | 7 (0.6) | 6.3×10^-2^ | ABCA1, abca4, ABCB4, abcc4, Abcg2, tap1, TAP2 |
| Jak-STAT signaling pathway | 16 (1.5) | 7.6×10^-2^ | IRF9, Ifna10, IFNA17, IFNA4, IFNB1, il2rb, il20ra, IL22RA1, IL23A, IL28B, IL29, IL6, IL7R, PIAS2, LOC344593, LOC442113, PTPN11, STAT1 |
| Viral myocarditis | 9 (0.8) | 8.7×10^-2^ | HLA-A, HLA-E, HLA-F, HLA-DOB, hla-dpa1, HLA-DRA, hla-drb1, HLA-DRB4, LOC652614, hla-dqa1, LOC100133678 |
| ECM-receptor interaction | 10 (0.9) | 9.3×10^-2^ | CD36, COL4A4, Col5a2, fn1, HMMR, Itgav, ITGB8, LAMA3, LAMC2, SDC2 |

## Slide 23
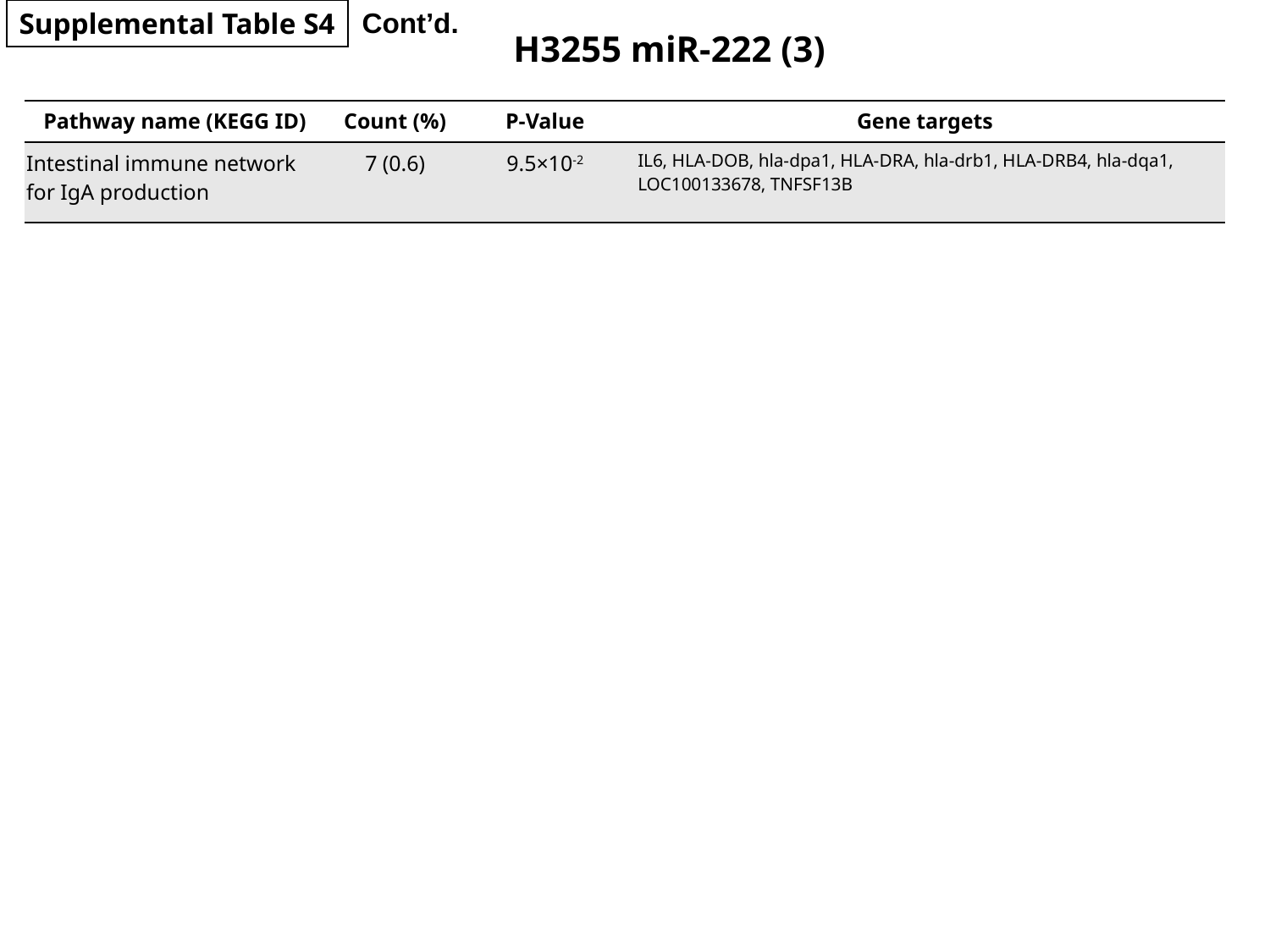

Supplemental Table S4
Cont’d.
H3255 miR-222 (3)
| Pathway name (KEGG ID) | Count (%) | P-Value | Gene targets |
| --- | --- | --- | --- |
| Intestinal immune network for IgA production | 7 (0.6) | 9.5×10^-2^ | IL6, HLA-DOB, hla-dpa1, HLA-DRA, hla-drb1, HLA-DRB4, hla-dqa1, LOC100133678, TNFSF13B |
